# Dynamics of Abundant and Rare Bacteria During Degradation of Lignocellulose from Sugarcane Biomass

**DOI:** 10.1007/s00248-019-01403-w

**Published:** 2019-07-08

**Authors:** Pilar Eliana Puentes-Téllez, Joana Falcao Salles

**Affiliations:** 1 Microbial Community Ecology, GELIFES, Groningen Institute for Evolutionary Life Sciences, University of Groningen, Nijenborgh 7, 9747 AG Groningen, The Netherlands; 2 Present Address: Department of Biology, Institute of Environmental Biology, Ecology and Biodiversity Group, Padualaan 8, 3584 CH Utrecht, The Netherlands

**Keywords:** Lignocellulose, Degradation, Abundant, Rare, Enrichment

## Abstract

Microorganisms play a crucial role in lignocellulosic degradation. Many enriched microbial communities have demonstrated to reach functional and structural stability with effective degrading capacities of industrial interest. These microbial communities are typically composed by only few dominant species and a high number of usually overlooked rare species. Here, we used two sources of lignocellulose (sugarcane bagasse and straw) in order to obtain lignocellulose-degrading bacteria through an enriched process, followed the selective trajectory of both abundant and rare bacterial communities by 16S rRNA gene amplification and analyzed the outcomes of selection in terms of capacities and specialization. We verified the importance of pre-selection by using two sources of microbial inoculum: soil samples from a sugarcane field with history of straw addition (St15) and control samples, from the same field, without amendments (St0). We found similitudes in terms of stabilization between the abundant and rare fractions. We also found positive correlations of both abundant and rare taxa (like Caulobacteraceae and Alcaligenaceae*)* and the degradation of lignocellulosic fractions. Differences in the inocula’s initial diversity rapidly decreased during the enrichment resulting in comparable richness levels at the end of the process; however, the legacy of the St15 inoculum and its specialization positively influenced the degradation capacities of the community. Analysis of specialization of the final communities revealed increased straw degradation capacity in the communities enriched in bagasse, which could be potentially used as a strategy for improving lignocellulose waste degradation on the sugarcane fields. This work highlights the importance of including the rare fraction of bacterial communities during investigations involving the screening and assessment of effective degrading communities.

**Electronic supplementary material:**

The online version of this article (10.1007/s00248-019-01403-w) contains supplementary material, which is available to authorized users.

## Introduction

Lignocellulose, the world largest agro-industrial by-product, is a complex substrate mainly composed of cellulose, hemicellulose, and lignin. The main agricultural lignocellulosic residues from sugarcane cropping are bagasse and straw. As part of sustainable practices, they are used as raw material for energy production (ethanol, methane, and other primary energy sources in the field) [1]. However, there is an overproduction that accumulates in landfills without any treatment that mostly ends up in burning [2]. An alternative strategy is to utilize straw as part of the cultivation practice by adding a straw blanket left on the ground—which can improve soil physical/chemical properties and protect it from erosion—reducing the environmental impact while improving sugarcane productivity [3].

Straw is, however, structurally complex and although microorganisms play a crucial role in lignocellulosic degradation in nature, their abundance in soils might constraint degradation rates. Moreover, biodegradation of lignocellulose is also regulated by the degradation capacity of microbial populations when working collectively [4, 5], through benefits like acquisition/exchange of metabolites and protection against environmental stress [6, 7]. Thus, the composition of the lignocellulose-degrading microbial community, as well as the relative abundances of the microbial components, determines the overall community functioning and associated degradation rates.

Strategies to identify species and/or to obtain microbial consortia that can effectively degrade agricultural waste have been developed worldwide. For instance, microbial consortia can be enriched by incubation of environmental samples—in the presence of lignocellulose—and successive transfers (sub-cultivation). This approach involves the stimulation of lignocellulose degraders from an initially high diverse community through the continuous enrichment of phenotypic adaptive traits, which leads to a reduction in diversity [8, 9]. Enrichment approaches provide an excellent method to generate lignocellulose-degrading communities, being widely used to obtain effective microbial communities capable of decomposing lignocellulosic biomass [5, 10–16].

Lignocellulose-degrading microbial consortia are, despite the reductionist approach from which they arise, still very complex and composed by few dominant species and many rare ones [5, 12], whose contribution to the degradation potential remains unclear. In natural settings, both abundant and rare populations are highly dynamic and are likely to play active roles [17–19]; however, the constant removal of rare microbial taxa from data sets [20] constrains our knowledge to the dominant species only. When put in the spotlight, rare microorganisms revealed to be relevant players of ecosystem function [20–22] and directly involved in the functional and structural stabilization of microbial communities through interactions with the abundant members [23, 24]. Thus, in the context of lignocellulose degradation, it is crucial to understand the relationship between diversity, including both the abundant and rare members, and the degradation capacity. Moreover, there is little information about the identity and ecological roles of the rare fractions of the consortia during lignocellulose degradation and whether the legacy and diversity of the inoculum used in these enrichment experiments influence the outcomes of selection for both rare and abundant fractions of the population. Altogether, this knowledge can highlight the importance of reconsidering both fractions in order to increase degradation yields at an industrial level.

Here, we investigated the dynamics of both abundant and rare members of lignocellulose-degrading microbial consortia, obtained through the enrichment procedure, using two soil inocula collected from a sugarcane plantation—from fields with or without straw amendments—and grown in two lignocellulosic sugarcane-related by-products (bagasse and straw). We followed and analyzed the trajectory of the community structure in each soil inoculum as well as community functioning (degradative performance) by using a 16S rRNA gene amplicon sequencing in combination with FTIR analysis of degradation. Thus, we sought to identify the abundant and rare taxa having positive or negative correlation with the degradation of lignocellulose. We then focused on investigating the final outcome of the enrichment process, in terms of capacities and possible ecological roles of both abundant and rare members, and on how the legacy of inoculum and specialization of the consortia influences the degradation potential of the community.

## Materials and Methods

### Inocula and Substrates

Soil samples from a sugarcane field near Piracicaba, SP, Brazil. Two soil samples (1000 g each, composite samples) obtained from nearby fields (same farm) were used as inoculum. One field has received sugarcane straw, which was left on the soil surface (15 Mg ha^−1^; soil “S15”) during 1.5 years as part of another study. The other soil inoculum came from an adjacent field without straw amendment (0 Mg ha^−1^; soil “S0”). Two different substrates obtained from sugarcane cultivation (i.e., bagasse and sugarcane straw) were used as lignocellulosic energy source. Bagasse and sugarcane straw were air-dried and grounded to a size of < 1 mm using a hammer mill.

### Enrichment Experiment

The experiments were performed in 100 mL flasks containing 25 mL of mineral salt medium (MSM; [25]) and with 1% of the grounded lignocellulose substrate (either bagasse or straw). Prior to inoculation, the flasks containing media were sterilized by autoclaving at 120 °C for 20 min. Inoculum consisted of soil suspensions, obtained by mixing 10 g of each soil with 90 mL of sodium chloride 0.90% and 10 g of sterile gravel in 250 mL flasks, which were shaken for 1 h at 250 rpm at room temperature (20 °C). A 4-mL sample of the soil suspension was stored at − 20 °C for DNA extraction of the inoculum. At the start of the enrichment, 250 μL of soil suspension was inoculated to the sterile lignocellulose medium (25 mL) containing either bagasse (B) or straw (St) as lignocellulos source, in triplicate (transfer flasks: T1), thus generating the following treatments: BS0, bagasse and inoculum from soil without amendment; BS15, bagasse and inoculum from soil amended with straw; StS0, straw and inoculum from soil without amendment; StS15, straw and inoculum from soil amended with straw. Additionally, two controls were included in triplicate, one with the substrate without inoculum and one with the inoculum without substrate. All the flasks were incubated at 28 °C, 180 rpm. During incubation, cell densities were verified microscopically at regular time intervals and when cultures reached 10^−9^ cells/mL (96 h), an aliquot of 25 μL of culture was transferred into 25 mL of fresh medium (transfer flasks: T2). This was repeated 10 times (T1 to T10). From T3, the time required to get such cell density was ~ 72 h. The enrichment experiment was performed in a total of 35 days. Cell counting of the two types of controls confirmed low levels of growth from the initial transfers. The number of cells in the control flasks without substrate decreased rapidly after T2 and arrived to < 10 cells/mL after T5. At each transfer, 2 mL samples were taken from each consortium, centrifuged and the pellet stored on 20% glycerol at − 20 °C for further DNA extraction.

### DNA Extraction

Total DNA extractions were performed from 2 mL samples collected from the consortia. Cells were centrifuged at 8000 rpm during 10 min and pellets were used for DNA extraction using UltraClean® Microbial DNA Isolation Kit (MoBio® Laboratories Inc., Carlsbad, USA) following the manufacturer’s instructions. Extracted DNA was used to performed DGGE analysis (see supplementary information, Fig. S1) and for sequencing analysis.

### 16S rRNA Sequencing and Bacterial Community Analyses

Sequencing of 16S rRNA gene was performed on purified DNA using the Illumina Miseq platform (Argonne National Laboratory, IL, USA). The 253-bp bacterial 16S rRNA gene amplicons were generated using the primer set 515F-806R. Reads were assigned to OTUs using an open-reference OTU picking protocol available in the QIIME 1.9.1 toolkit [26]. UCLUST [27] was applied to search for sequences against a subset of the Greengenes 13.8 database [28] filtered at 97% identity and followed by a selection of representative sequences. Analyses of community structure, as well as richness and diversity estimators, were carried out at a depth of 14,000 bacterial rarefied sequences per sample, to eliminate the effect of sampling effort. Although this rarefaction might exclude a fraction of rare taxa, we decided to compare all samples from different origins and substrates across the same sequencing depth. We identified and removed chimeras via ChimeraSlayer and subsequently excluded chimeric sequences from the main OTU table. We used Greengenes 13.8 database in order to obtain low-rank taxonomic classifications (family and genus) [29]. QIIME was also used to generate alpha- and beta-diversity metrics, including OTU richness, phylogenetic diversity (PD), and UniFrac distances. For all multivariate analyses, we used Primer 6 with the add-on package PERMANOVA+ (PRIMER-E Ltd., Plymouth, UK). Abundant and rare taxa were defined arbitrarily using a cutoff of 2% relative abundance (abundant > 2%; rare < 2%) [29–31]. Since the cutoff used to define rare taxa may affect the main results presented in this study, we tested the cutoff 1%. The classified taxa were used to observe the contribution of specific taxa to the dissimilarities between substrates and soil origins (using SIMPER (PRIMER-E)).

### FTIR Analysis

For the calibration set, pure cellulose (microcrystalline powder), hemicelluloses (xylan from birch wood), and lignin (hydrolytic) powders were obtained from Sigma-Aldrich Canada Ltd. (St. Louis, MO), and were subsequently mixed in different proportions (Supplementary information Table S1) to determine the relationship between their respective quantity in the mixture and representative Fourier transform infrared spectroscopy (FTIR) spectra [32]. Particle size of both the calibration set and samples was defined with a 106 μm sieve. Spectra were recorded using a Perkin-Elmer VATR TWO spectrometer (Waltham, MA, USA) in the wavenumber range of 800–1800 cm^−1^ with a resolution of 4 cm ^−1^ under ambient atmosphere, at room temperature and in triplicates. The spectra were integrated and baseline corrected using the Spectrum ™ software. The analysis was performed using the Unscrambler X (Camo Software, Oslo, Norway). A 5-point Savitzky–Golay smoothing algorithm was applied to the calibration set’s spectra and used to predict concentrations in the samples (using partial least squares regression (PLS)). The predicted composition of each sample obtained with PLS was expressed as the percentage of degradation (%*D*) and was calculated for each lignocellulose component of the lignocellulose as follows: %*D* = [(*a* − *b*) / *a*] × 100; where *a* = percentage of the component in the substrate before incubation; *b* = percentage of the component in the substrate after incubation. Initial amounts of each lignocellulosic component in the untreated substrate (lignin, hemicellulose (xylan), and cellulose) were determined and considered as 100%; thus, %*D* was interpreted as the amount (in percentage) consumed by the microbial activity from the available substrate.

Statistical comparisons between degradation percentages were performed using *t* test and one-way ANOVA (Tukey’s test). Linear correlations between α-diversity measurements and abundant and rare microbial communities and %*D* were obtained with IBM SPSS Statistics for Macintosh, Version 24. Armonk, NY: IBM Corp. Redundancy analysis (RDA) was performed to explore the linear relationship between both abundant and rare taxonomic groups and the environmental variables (lignocellulosic components) using Canoco software v5.0 (Wageningen, The Netherlands) [33].

## Results

### Trajectory of Diversity and Structure of Bacterial Communities Across the Enrichment Experiment

**Fig. 1:**
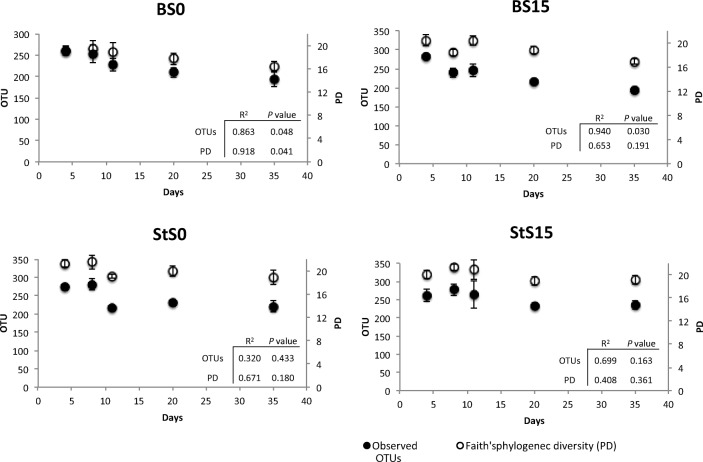
α-diversity measurements during the enrichment richness (number of OTUs) and Faith’s phylogenetic diversity (PD) were measured using rarefied sequences at a depth of 14,000 sequences. α-diversity measurements were calculated from the OTU information obtained using QIIME 1.9.1. Bars refer to standard errors (*n* = 3). *X* axis indicates the number of days in the enrichment process. 4 days = T1; 8 days = T2; 11 days = T3; 20 days = T6; 35 days = T10. In a box: the result (*R*^2^ and *P* value) of the linear regression analysis on PD and OTU data

Bacterial 16S rRNA from both inocula (S0 and S15) as well as from selected transfers from the enrichment cultures (T1, T2, T3, T6, T10; sample selection based on stabilization patterns revealed by DGGE analyses of all transfers, see supplementary information; Fig. S1) and controls were used to determine eventual changes in the community α-diversities across the enrichment. Analysis at a sequencing depth of 14,000 reveals differences and a great variability of diversity across all samples (Supplementary information Fig. S2). From this analysis, we found a significant difference (*P* < 0.05) in the number of OTUs and phylogenetic diversity index between both initial inocula S0 (625 OTUs and PD = 58.9% averaged) and S15 (515 OTUs and PD = 52.4% averaged). As expected, incubation on substrate leads to a significant reduction in the initial diversity (OTU richness and PD, see Table 1), which was more pronounced for samples inoculated with S0 than S15. Linear regression analysis of both α-diversity measurements (OTU number and PD) across the enrichment time (Fig. 1) indicated that the number of OTUs continued to decrease significantly only in bagasse-treated samples (*P* < 0.05) whereas significant change in PD at the initial stages of enrichment was observed only for BS0 samples (*P* < 0.05). Pairwise comparisons between time points indicate that after T3 (11 days of culture), significant differences in richness values between samples were no longer present. This shift in community composition continued relatively constant until the end of the enrichment (Table 2).

**Table 1:** Average percentage of α-diversity values per sample type

Sample	OTU richness (%)	PD value (%)
BS0	69.2	72.1
BS15	62.3	67.6
StS0	64.7	68
StS15	54.3	63.4

**Table 2:** *Paired-wise* comparison between transfers (by soil type) along the enrichment. In italics: results with significant difference

Soil type	Groups	*P*(perm)
S0	*S0T1*, *S0T2*	*0.001*
	S0T2, S0T3	0.771
	S0T3, S0T6	0.949
	S0T6, S0T10	0.992
S15	*S15T1*, *S15T2*	*0.001*
	S15T2, S15T3	0.64
	S15T3, S15T6	0.517
	S15T6, S15T10	0.817

**Fig. 2:**
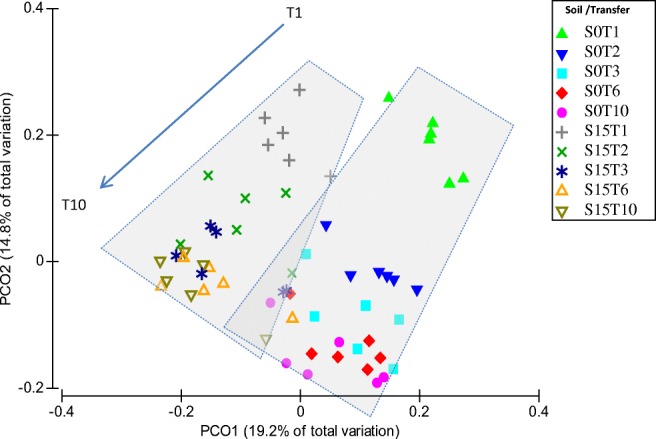
Principal coordinate analysis (PCoA) of unweighted-UniFrac distances of all samples. Filled symbols represent S0 samples. Non-filled symbols represent S15 samples. Dashed boxes and arrow represent the separation and direction of the trajectories of the two soil types along the enrichment experiment, respectively

Principal coordinates analysis (PCoA; using unweighted-UniFrac distance), used to analyze the trajectories of phylogenetic β-diversity, showed a tendency of separation according to soil inocula (S0 or S15) (PERMANOVA Pseudo-F 3.27; *P*(perm) < 0.001) and not to substrate (Fig. 2), with an explained total variation of 34%.

### Variations of the Most Abundant Taxonomic Groups during the Enrichment Experiment

**Fig. 3:**
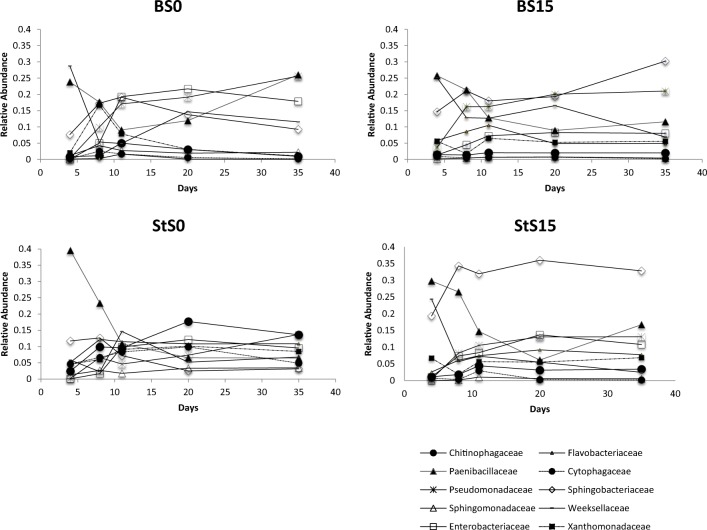
Relative abundance at family level of the most abundant taxa (relative abundance > 2%) along the enrichment experiment based on 16S rRNA gene amplicon sequence. *X* axis indicates the number of days in the enrichment process. 4 days = T1; 8 days = T2; 11 days = T3; 20 days = T6; 35 days = T10. Data is organized by substrate (B, bagasse; St, straw) and soil types (S0 and S15)

We determined the most abundant families (top abundant) having a relative abundance > 2% at least once during the enrichment process and the “rarest” families (top rare) having relative abundances < 2% during the enrichment. We found no differences in the taxa classified as most abundant and rare at the same sequencing depth (all rare taxa have relative abundances < 1%, see supplementary information Table S2). Analysis of the community structure in T1, T2, T3, T6, and T10 reveals progressive changes in the dynamics of taxonomic groups reaching abundance levels > 2% at least once during the enrichment process (Fig. 3). We could observe an influence of the type of substrate on the dynamics of the most abundant groups. Specifically, we noticed a more dynamic behavior of abundant taxa in the bagasse treatment compared to the straw treatment (*P* < 0.05), the latter showing rapid stabilization of abundant taxa. We obtained low-rank taxonomic information from the computational analyses (family and genus level). In order to facilitate comparisons across samples, we based our taxonomic analyses at the family level. We are aware of the variations at the genus or species level within family members. Using Greengenes13.8, we looked at the genus level in the families with outstanding results across the experiment. Although fluctuating, relatively high levels of Paenibacillaceae (*Paenibacillus* sp.), Pseudomonadaceae, Enterobacteriaceae, and Sphingobacteriaceae remained present in bagasse samples until the end of the enrichment. Paenibacillaceae (*Paenibacillus* sp.), which shows an outstanding initial high abundance in all samples, rapidly decreased across all samples, except for a particular recovering trend observed in the second part of the experiment in BS0 samples (final relative abundance T10 [FRA] = 26.0 ± 3%); the highest observed in these samples). The second most abundant taxa in all bagasse samples were Pseudomonadaceae (FRA = 23.2 ± 0.033%) and Enterobacteriaceae (FRA = 12.9 ± 0.07%). Particularly, the most abundant taxa at the end of the experiment in BS15 samples belonged to Sphingobacteriaceae (FRA = 30.2 ± 12%).

On the other hand, StS0 and StS15 samples carried relatively high levels (in a range of 8 to 14%) of Pseudomonadaceae, Paenibacillaceae, Enterobacteriaceae, Flavobacteriaceae, and Chitinophagaceae during the experiment. StS15 samples, however, showed outstanding levels of Sphingobacteriaceae (FRA = 32.8 ± 9%), which started early (from T2) and were kept until T10. Other taxonomic groups like Xanthomonadaceae, Weeksellaceae (*Chryseobacterium* sp.), and Cytophagaceae maintained steady and similar levels among the abundant taxa in all straw samples.

In general, even though there was a clear difference of the trajectories per substrate type, we could observe similarities of the most abundant taxa per soil origin, especially in S15 samples, where the top three most abundant family groups were the same: Sphingobacteriaceae, Pseudomonadaceae, and Paenibacillaceae. SIMPER analyses comparing soil and substrate types and performed with the data from the most stable (in terms of diversity, T10) moment during the enrichment revealed that most of the contribution to the Bray–Curtis dissimilarity between soil origin (S0 or S15) and substrates (bagasse or straw) was driven by Sphingobacteriaceae (*Sphingobacterium* sp*.* across all time points), which accounted for 18.47% in soil type and 8.8% in substrate.

### Dynamics of the “Rare Taxonomic Groups”

**Fig. 4:**
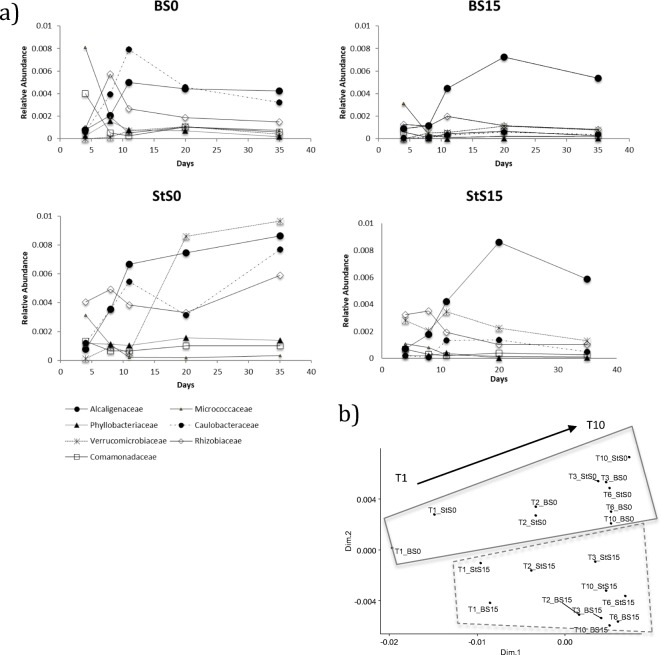
**a** Relative abundance at family level of the top rare taxa (relative abundance < 2%) along the enrichment experiment based on 16S rRNA gene amplicon sequence. *X* axis indicates the number of days in the enrichment process. 4 days = T1; 8 days = T2; 11 days = T3; 20 days = T6; 35 days = T10. **b** MDS displaying the trajectory of the rare members of the communities along the enrichment. The arrows indicate time and trajectory. Data is organized by substrate (B, bagasse; St, straw) and soil types (S0 and S15)

In much the same way as the most abundant taxonomic groups were analyzed, we zoomed in into the dynamics and identity of the less abundant (rare) taxonomic groups (relative abundance < 2% during all the enrichment; Fig. 4a). A MDS on the rare-family taxonomic groups shows stabilization of these communities after T3 (Fig. 4b) driven by soil type, which resembled the stabilization pattern driven by the abundant fraction (Fig. 2). We identified the top rare families having a relative abundance < 1%. Analysis of similarity (ANOSIM) from the top rare families indicates high separation of these rare groups according to soil origin (*P* < 0.05; *R*^2^ = 0.872) and not to substrate type (*P* > 0.05). S0 samples, for example, have predominant and steady levels of the families Alcaligenaceae (*Achromobacter* sp.), Verrucomicrobiaceae, Caulobacteraceae (*Caulobacter* sp., *Mycoplana* sp., *Phenylobacterium* sp.), Rhizobiaceae (like *Kaistia* sp.), and other rare organisms belonging to the families Micrococcaceae, Comamonadaceae, and Phyllobacteriaceae.

S15 analysis revealed comparable dominant family groups with S0 at the end of the enrichment. However, the difference between them is due to distinct and higher abundant levels of Caulobacteraceae (0.008% ± 0.0021) and Verrucomicrobiaceae (0.012% ± 0.0015) in S0 and outstanding levels of Alcaligenaceae in S15 samples (0.0059% ± 0.0036). SIMPER analysis of the top 20 rare families revealed that Caulobacteraceae contributed to the Bray–Curtis dissimilarity between S0 and S15 by a 25.3%, followed by high levels of Verrucomicrobiaceae (23.4%) and Alcaligenaceae (13.8%). Interestingly, we observed a higher fluctuation in the relative abundance of the rare families in samples receiving the S0 as inoculum as compared to S15, indicated by the trajectories in Fig. 4a and by the shift in community structure observed in Fig. 4b. Together, these results indicate that soil legacy might have already led to a stabilization of the less abundant families in S15.

### Dynamics of Lignocellulose Degradation during the Enrichment Experiment

The percentage of degradation (%*D*) of the three lignocellulosic components was calculated with the data obtained from FTIR analysis. Based on this data, degradation was obtained for each of the selected transfers (T1, T2, T3, T6, and T10). Untreated (without bacteria) bagasse was found to have a composition of 22.8% lignin, 39.37% cellulose, and 23.12% hemicellulose whereas untreated straw had a composition of 20.18%, 35.85%, and 20.12%, respectively. Overall, the compositional analysis showed that the contents of cellulose, hemicellulose, and lignin were in agreement with the ranges previously described in the literature [34].

**Fig. 5:**
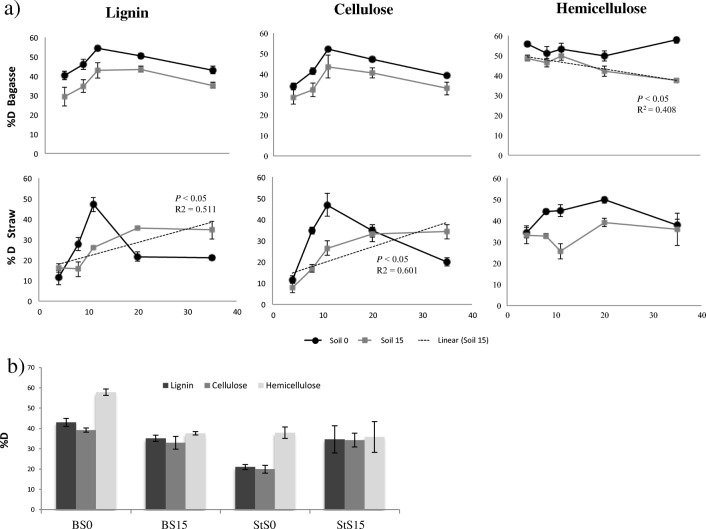
**a** Degradation patterns of each lignocellulosic fraction along the enrichment experiment; *P* and *R*^2^ values are indicated when linear regression is significant. **b** Comparison of the final degradation values (T10) across all samples

Patterns of degradation differed per soil origin and per substrate. The degradation results obtained with FTIR along time (Fig. 5a) revealed positive correlations between the degradation of lignin and cellulose for StS15 and a negative linear relationship for hemicellulose in BS15. For the remaining samples, the degradation levels remained steady along time, finishing the experiment with rather similar degradation levels to initial ones, except for an outstanding peak of degradation observed in T3 for most of the enrichments (Fig. 5a; Supplementary information Table S3).

The hemicellulose degradation showed an interesting pattern in bagasse samples. Initial levels of hemicellulose degradation in both BS0 and BS15 samples were the highest across all samples. Interestingly, whereas BS0 slightly increased degradation (which at the end of the experiment is the highest of all; Fig. 5b), variation of BS15 results strongly fitted a decreasing linear model for hemicellulose degradation (*R*^2^ = 40.8%; *P* = 0.010). Interestingly, the correlation between α-diversity measurements (number of OTUs and PD) and degradation of each lignocellulosic fraction along the experiment revealed no pattern, except for a positive significant correlation between these measurements and the degradation of hemicellulose in BS15 samples (*P* < 0.05) (Supplementary information Table S4). On the other hand, our straw-treated samples StS0 and StS15 have a different pattern of degradation along the enrichment. Whereas in StS0 the degradation of all three lignocellulosic fractions did not significantly change during the enrichment (despite the T3 peak), in StS15 samples we observed a significant improvement in the degradation capacities of lignin (*R*^2^ = 51.1%; *P* < 0.05) and cellulose (*R*^2^ = 60.1%; *P* < 0.05). When analyzing the percentages of improvement between initial levels of degradation versus final degradation, we observed an increase of only 8% and 4% in degradation levels across all lignocellulosic fractions in BS0 and BS15, respectively, but an outstanding improvement in the degradation levels of StS0 (55%) and StS15 (153% = 1.5 times higher).

### Correlation between Abundant and Rare Communities and Degradation Capacities

In order to find the strength and nature (positive or negative) of any correlation between taxa (abundant and rare) with the degradation along the enrichment experiment, we calculated *R*^2^ values and their significance through linear regression models (Table 3) between these two data sets. We then noticed several taxa with strong and significant correlations (*R*^2^ > 70%).

**Table 3:** *R*^2^ values obtained from the linear regression between each lignocellulosic fraction and the most abundant and predominantly rare members. Bold and italics indicate strong and significant correlation (*R*^2^ > 70% (cutoff arbitrarily chosen); *P* < 0.05). Bold: positive correlation; italics: negative correlation (negative slope)

	BS0			BS15			StS0			StS15		
	Lignin	Cellulose	Hemicellulose	Lignin	Cellulose	Hemicellulose	Lignin	Cellulose	Hemicellulose	Lignin	Cellulose	Hemicellulose
Abundant families												
Chitinophagaceae	**0.88**	**0.87**	0.15	0.52	0.55	0.12	0.02	0.17	**0.73**	0.47	0.63	0.1
Cytophagaceae	0.2	0.18	0.3	0.03	0.004	0.49	0.25	0.62	**0.89**	0.007	0.0001	*0.78*
Enterobacteriaceae	0.49	0.56	0.02	0.67	0.52	0.34	0.16	0.24	0.35	**0.72**	**0.87**	0.17
Flavobacteriaceae	0.58	0.57	0.01	0.06	0.17	0.51	0.42	0.59	0.5	0.68	**0.88**	0.06
Paenibacillaceae	*0.9*	*0.84*	**0.7**	0.7	0.57	0.31	0.21	0.29	0.37	*0.8*	*0.78*	0.07
Pseudomonadaceae	0.17	0.24	0.005	0.47	0.29	0.44	0.0024	0.003	0.03	0.68	**0.92**	0.05
Sphingobacteriaceae	**0.71**	0.62	0.42	0.0008	0.08	0.74	0.003	0.02	0.13	0.31	0.59	0.03
Sphingomonadaceae	0.09	0.09	0.18	**0.99**	**0.88**	0.01	0.01	0.008	0.19	0.4	0.52	0.14
Weeksellaceae	0.44	0.48	0.11	0.15	0.1	0.33	0.52	0.22	0.001	0.37	*0.69*	0.01
Xanthomonadaceae	0.07	0.05	0.29	0.07	0.16	0.0001	0.23	0.36	0.49	0.23	0.05	0.01
Rare families												
Alcaligenaceae	0.58	0.65	0.01	0.61	0.47	0.34	0.15	0.17	0.22	**0.9**	**0.83**	0.25
Caulobacteraceae	**0.9**	**0.93**	0.15	**0.66**	0.5	0.31	0.18	0.05	0.0009	0.46	0.41	0.0056
Comamonadaceae	0.44	0.48	0.08	0.05	0.07	0.03	*0.72*	*0.76*	0.36	0.2	0.51	0.07
Micrococcaceae	0.48	0.54	0.08	0.57	0.43	0.17	0.37	0.5	0.51	*0.88*	*0.97*	0.03
Phyllobacteriaceae	0.15	0.12	0.56	0.65	0.64	0.01	0.28	0.06	0.07	0.23	0.12	*0.96*
Verrucomicrobiaceae	0.61	0.63	0.23	0.6	0.36	0.4	0.07	0.02	0.03	0.13	0.15	0.5
Rhizobiaceae	0.06	0.04	0.3	0.2	0.41	0.62	0.02	0.12	0.23	*0.98*	*0.86*	0.13

In the bagasse enrichments, the abundant taxa with a strong and positive relationship with the degradation of lignin and cellulose in BS0 were Chitinophagaceae and Sphingobacteriaceae (*P* < 0.05). Interestingly, Caulobacteraceae (characterized as rare) had also a strong positive correlation with these two fractions. We also found a negative correlation (*P* < 0.05) with the abundance of Paenibacillaceae with the degradation of these two fractions in BS0 samples; however, a positive correlation was observed between the abundant levels of this taxon and hemicellulose degradation. Furthermore, the steady levels of lignin and cellulose degradation in BS15 samples were only positively correlated with the abundant levels of Sphingomonadaceae (*P* < 0.05), which explained a large percentage of variation in both fractions, given the extremely high *R*^2^ values (Table 3). A positive correlation was also observed for the rare taxa Caulobacteraceae found in BS15 samples and lignin (*P* < 0.05).

For the samples enriched in straw and S0 inoculum (StS0 samples), we found few correlations between taxa and the steady levels of degradation. We only found a positive correlation of hemicellulose degradation with the abundant levels of Chitinophagaceae and Cytophagaceae (*P* < 0.05). Instead, the presence of Comamonadaceae (rare taxa during the enrichment) had a negative relationship with lignin and cellulose (*P* < 0.05). Conversely, several abundant and rare taxa were positively linked to increased lignin and cellulose degradation in StS15. We found here positive and significant (*P* < 0.05) correlations with the abundance of Enterobacteriaceae, Flavobacteriaceae, and Pseudomonadaceae and the rare Alcaligenaceae (*R*^2^ > 80%, *P* < 0.05). Negative relationships with the degradation of these two fractions were found in the abundant Paenibacillaceae and the scarce levels of Micrococcaceae and Rhizobiaceae (*P* < 0.05). Hemicellulose was negatively linked to the abundant levels of Cytophagaceae and the rare Phyllobacteriaceae (*P* < 0.05)*.*

We then used RDA analyses to depict the overall relationship between abundant and rare taxonomic groups with degradation during the stable stage of the experiment (T6 and T10; supplementary information Fig. S3). Preliminary detrended correspondence analysis (DCA) of both communities revealed that the longest gradient lengths were shorter than 3.0, confirming that the majority of family groups exhibited linear responses to the lignocellulosic components’ variation [35, 36].

The significance of the correlation between degradation and the communities was evaluated by Monte Carlo permutation test (999 permutations) and did not show significant values for the community abundance in T1, T2, and T3 (*P* > 0.05) for both types of data sets (abundant and rare). T6 and T10 abundant communities, on the other hand, were significantly explained by the lignocellulose components (pseudo-F 2.8, *P* = 0.02), in particular by lignin and hemicellulose (*P* < 0.05). Furthermore, the summarized effect of the explanatory variables revealed a significant correlation between the lignin degradation and the rare taxa present in T6 and T10 (*P* < 0.05).

### Specificity of the Communities—Crossed Experiment

**Fig. 6:**
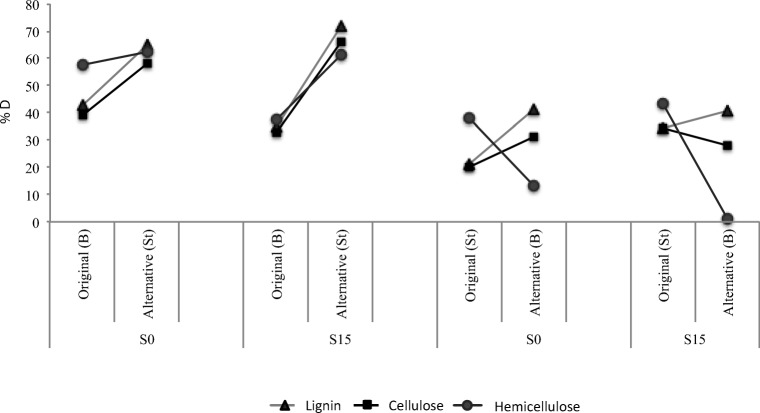
Degradation patterns of each lignocellulosic fraction during the crossed experiment. Original degradation capacities for each lignocellulosic component (original T10 value obtained in the enrichment experiment) and results obtained in the new (alternative) tested environment

Due to differences found in the degradation capacities according to the substrate, we decided to test the strength of their specificity by growing each of the T10 communities in a one-batch alternative substrate and record the resulting degradation values. The original (from the enrichment experiment, T10) degradation capacities in each lignocellulosic component and the new (alternative) tested environment by soil type (Fig. 6) revealed that enriched-in-bagasse communities (S0 and S15) degrade lignin and cellulose and hemicellulose significantly better in straw (*t* test *P* < 0.05). On the other hand, S0 communities coming from straw show roughly similar patterns when performing in bagasse. The only significant changes observed in the S0 samples coming from straw were an improvement in the degradation of lignin (*t* test *P* < 0.05) and a decline in the degradation of the hemicellulose fraction of bagasse (*t* test *P* < 0.05).

## Discussion

### Unstable and Stable Phases of Diversity During the Enrichment Experiment Correlate with the Dynamics of Degradation Capacities

In this study, two different soil inocula, one initially less diverse than the other, were exposed to the main sugarcane by-products in order to assess the correlation between community structure (abundant and rare members), the dynamics of degradation along an enrichment process, the effects of diversity, and the outcomes of selection in terms of degradation capacity. Although we are aware of the active part of fungi on the lignocellulose degradation, we sought to keep the focus of this study on bacterial communities because of their primary role in degrading lignocellulose material. In addition, because it has been found that this type of enrichment maintains rather low and consistent levels of fungi [12, 25].

The DGGE and α-diversity analyses based on amplicon sequencing allowed us to divide the overall trajectory of the enrichment in two parts: a first half (T1, T2, T3) when communities are going through an unstable period and the environmental pressure (given by the substrate) is acting upon them and a second part where the communities where there is a more stable degradative capacity and diversity of the selected members (T6 and T10). In the first part, we noticed a higher reduction in the diversity levels in the samples containing bagasse which can be explained by the pre-adapted to straw condition of bacterial communities found in the straw samples. At this stage, the degradation competences start rising and are probably linked to the number of players, by T3 (11 days) they reached a degradation peak which is perhaps linked to the contemporary taxa’s interactivity. After T3, the communities arrive to a point where the overall degradative capacity and diversity of the selected members is more stable. Although this stabilization phase occurred early when compared to other similar studies [12, 25], we speculate that this can be explained by the legacy of our soil samples—from sugarcane fields origin—and therefore pre-selected for these substrates. The fact that stabilization of S0 communities occurred at a slower pace than in S15 strengthen our hypothesis. Furthermore, we noticed an initial effect of the substrate type, which by the end of the experiment was influenced by soil origin.

Our results from the crossed experiment further confirm that pre-treatment practices such as the use of straw layer on the sugarcane fields have an impact on microbial diversity and associated degradation capacities by stimulating the selection of effective straw-degrading bacteria, which developed a better capacity to digest all three lignocellulosic fractions from straw. This suggests a positive effect of a pre-treatment practices involving the application of straw or bagasse onto the field.

When comparing across all degradation results, the highest improvement in degradation capacity was observed in StS15 samples. All in all, a less diverse and pre-adaptive condition might have been stimulating to rapidly select effective forms and thus reach higher degradation levels. On the contrary, the fate of StS0 communities was rather unfavorable in terms of degradation. Altogether, we can conclude here that the initial richness was determinant to the degradative outcome, its variability, and the effect of the type of substrate as initial selecting driver.

### Rare Communities Resemble the Trajectories of Abundant Communities and Have Positive Correlations with Degradation

Analysis of bacterial communities using 16S RNA amplicon sequencing could include several biases related to gene copy numbers and database updates; however, this approach is still widely used to reveal structural composition and richness in environmental samples [37] and can be used as a proxy to depict the most abundant and rare fractions. Overall in our results, the legacy and diversity of the inoculum used influenced the outcomes of selection for both rare and abundant families. However, we observed an exclusive impact of the type of substrate on the abundant communities. We also noticed similarities of the rare-communities stabilization patterns with the stabilization patterns of abundant communities. These results strongly suggest the presence of interactive relationships between abundant and rare microorganisms. For example, rare microorganisms could take care of a specific function with a direct action on the substrate or induce metabolic responses in more abundant microbes, or they can be acting as waste product consumers or as facilitators of growth, implying that rare microbes have direct as well as indirect effects on ecosystem functioning [20].

From correlation analyses, we can suggest positive as well as negative relationships of abundant and rare taxa with degradation with bacteria belonging to few phyla (*Bacteroidetes*, *Proteobacteria*, *Firmicutes*, and *Verrucomicrobia*). However, a more quantitative approach (e.g., PCR base methods or FISH) is necessary to validate functional roles in the degradation process. The rather steady degradation results of lignin and cellulose from bagasse samples could have been supported by the dynamic behavior of abundant lignocellulose-degrading taxa like Chitinophagaceae (*Bacteroidetes* phylum) and Sphingomonadaceae (*Proteobacteria* phylum) [38–43]. Interestingly, Brazilian soils with sugarcane culture have been found to carry many species of the *Chitinophagaceae* family able to degrade lignocellulose [38], although their specific role in degradation is still unclear [25]. Interestingly, Paenibacillaceae (*Firmicutes* phylum), which has been found to be active lignin and hemicellulose degraders [12, 44], was linked to the high degradation levels of hemicellulose degradation in BS0 samples. This taxon showed a recovering trend in the second part of the experiment in these samples with a high relative abundance value. This suggests an active role of this taxon in the degradation of this fraction. We also noticed a positive relationship (although not statistically significant) between the relative abundance of Sphingobacteriaceae (*Sphingobacterium* sp.; *Bacteroidetes* phylum), with the maintained (high) degradation levels of BS0 treatment. This family has been found to carry enzymatic capacities to degrade lignin [45] and hemicellulose fractions of wheat straw [46] but also as acting like “cheaters” during the degradation process, helping to remove the cello-oligosaccharides produced by polymer degraders [25].

Among the most abundant taxa in straw samples were Pseudomonadaceae (*Proteobacteria* phylum), Chitinophagaceae, and Flavobacteriaceae (both *Bacteroidetes* and the latter known to have effective lignocellulolytic activities [46–49]), having a positive correlation with the degradation levels at the end of the enrichment. Interestingly, even though the role of Chitinophagaceae in degradation is unknown [25], here we found positive correlation of these taxa with the degradation of hemicellulose along the enrichment. The highest improvement in degradation was obtained in STS15 samples and it was supported by the presence of a group of several effective lignocellulosic degraders: Enterobacteriaceae (*Proteobacteria* phylum), Flavobacteriaceae, Pseudomonadaceae [25, 39, 46, 48]. This set of microorganisms might have entered into a “division of labor” dynamics, not only by unlocking the substrates but also by consuming each other’s metabolic products. Sphingobacteriaceae was found to be highly abundant in these samples at the end of the enrichment, which as “cheater” player might be also contributing to the optimal degradation process by consuming by-products produced, by the active degradation players.

Furthermore, our results demonstrated the importance of rare communities in lignocellulosic degradation. We observed a comparable trajectory of rare communities with the abundant ones in terms of stabilization patterns. These results suggest the presence of interactive roles (either positive or negative) of these communities with the dominant ones and a direct contribution to the overall community functioning. Therefore, it becomes critical to assess the contribution of rare bacteria towards specific functions in light of the high diversity of bacterial communities [24]. The maintenance of both abundant and rare can be explained from physiological aspects like slower growth rates and specific metabolic actions (like contributing to nutrient scavenging) and other supportive and interactive activities. Recent genomic evidence indicates that rare communities add the capacity to rapidly respond to environmental changes and presumably become abundant in specific situations [24].

In this study, specific rare taxa were found to have positive correlations with degradation. For example, we found a strong and positive correlation of the low (rare) levels of Caulobacteraceae (*Proteobacteria* phylum) with the highly maintained degradation of lignin and cellulose of bagasse-treated samples. The presence of these taxa in sugarcane production systems has been reported [42] as well as their lignin degradation capacities [50]. For example, DeAngelis et al. [41] reported abundant levels of *Caulobacter* types (catalase producers) in lignin-amended soils. In another notice, we found the rare Comamonadaceae (*Proteobacteria* phylum) having a negative effect in degradation in StS0 samples. This family group has the ability to metabolize complex organic compounds as energy sources for growth [51].

One of the most predominant rare taxa in the best-performance community (STS15) was Alcaligenaceae (*Achromobacter* sp.; from *Proteobacteria* phylum). The ability to use complex carbohydrates has not been described for the genus *Achromobacter* sp. [52, 53]. However, other members of the Alcaligenaceae family have been previously reported in lignocellulose-degrading composite systems [54]. The endurance of *Achromobacter* sp. within lignocellulosic consortia could be due to their ability to utilize simple sugars produced by potent hydrolytic strains [55], which enhanced and actively supported the dynamics of a very active group of abundant set of microorganisms found in these samples. A direct functional validation could be used in the future to confirm these findings.

Among the rare fractions of low performance enrichment, we found effective degraders of lignocellulosic components like Verrucomicrobiaceae (*Verrucomicrobia* phylum) and Comamonadaceae [56–59]. We hypothesize that either their effect on the overall performance was not positive enough to reach high degradation levels or the persistent high diversity of the rare members did not allow an increase in the community’s performance.

A more quantitative study including the effect of specific rare taxa on the overall performance of the community would bring interesting insights on their specific ecological roles and the interactive relationships in the community. In this study, we revealed the dynamics of the rare fraction in an enrichment community using a 16S rRNA gene sequencing approach and observed a direct correlation with the abundant fraction. We found active and positive roles of the rare fraction of the communities in lignocellulose degradation. These results suggest a possible interplay between both abundant and rare communities. We demonstrated that the legacy and diversity of the inoculum positively influenced the outcomes of selection for both rare and abundant families. Furthermore, we found that communities evolved to digest a complex substrate like bagasse developed an improved capacity to unlock easily accessible substrates, which should be further investigated as a potential solution of biowaste treatment on the sugarcane fields. Altogether, here we highlight the importance of including the rare fraction during investigations directed to study the degradative performance of microbial communities seeking to increase biodegradation yields as well as the practice of applying a layer of straw on the fields as a pre-adaptive process as part of biodegradation practices.

## Electronic Supplementary Material

ESM 1 (PPTX 786 kb)

## References

[1] Hofsetz K, Silva MA (2012). Brazilian sugarcane bagasse: energy and non-energy consumption. Biomass Bioenergy.

[2] Vega-baudrit J, Delgado-Montero K, Madrigal-Carballo S (2011). Biodegradable polyurethanes from sugar cane biowastes. Cellul Chem Technol.

[3] Leal MR, Galdos MV, Scarpare F, Seabra JEA, Walter A, Oliveira C (2013). Sugarcane straw availability, quality, recovery and energy use. Biomass Bioenergy.

[4] Puentes-Tellez P, Salles J (2018). Construction of effective minimal active microbial consortia for lignocellulose degradation. Microb Ecol.

[5] Cortes-Tolalpa L, Jiménez DJ, de Lima Brossi MJ, Salles JF, van Elsas JD (2016). Different inocula produce distinctive microbial consortia with similar lignocellulose degradation capacity. Appl Microbiol Biotechnol.

[6] Waldrop MP, Balser TC, Firestone MK (2000). Linking microbial community composition to function in a tropical soil. Soil Biol Biochem.

[7] Warnecke F, Luginbühl P, Ivanova N, Ghassemian M, Richardson TH, Stege JT, Cayouette M, McHardy AC, Djordjevic G, Aboushadi N, Sorek R, Tringe SG, Podar M, Martin HG, Kunin V, Dalevi D, Madejska J, Kirton E, Platt D, Szeto E, Salamov A, Barry K, Mikhailova N, Kyrpides NC, Matson EG, Ottesen EA, Zhang X, Hernández M, Murillo C, Acosta LG, Rigoutsos I, Tamayo G, Green BD, Chang C, Rubin EM, Mathur EJ, Robertson DE, Hugenholtz P, Leadbetter JR (2007). Metagenomic and functional analysis of hindgut microbiota of a wood-feeding higher termite. Nature.

[8] Zuroff TR, Curtis WR (2012). Developing symbiotic consortia for lignocellulosic biofuel production. Appl Microbiol Biotechnol.

[9] Gao ZM, Xu X, Ruan LW (2014). Enrichment and characterization of an anaerobic cellulolytic microbial consortium SQD-11 from mangrove soil. Appl Microbiol Biotechnol.

[10] Brethauer S, Studer M (2014). Consolidated bioprocessing of lignocellulose by a microbial consortium. Energy Environ Sci.

[11] Wang W, Yan L, Cui Z, Gao Y, Wang Y, Jing R (2011). Characterization of a microbial consortium capable of degrading lignocellulose. Bioresour Technol.

[12] De Lima Brossi MJ, Jiménez DJ, Cortes-Tolalpa L, van Elsas JD (2016). Soil-derived microbial consortia enriched with different plant biomass reveal distinct players acting in lignocellulose degradation. Microb Ecol.

[13] Wongwilaiwalina S, Rattanachomsria U, Laothanachareona T, Eurwilaichitra L, Igarashib Y, Champredaa V (2010). Analysis of a thermophilic lignocellulose degrading microbial consortium and multi-species lignocellulolytic enzyme system. Enzym Microb Technol.

[14] Moraïs S, Shterzer N, Lamed R, Bayer E, Mizrahi I (2014). A combined cell-consortium approach for lignocellulose degradation by specialized *Lactobacillus plantarum* cells. Biotechnol Biofuels.

[15] Mohanram S, Amat D, Choudhary J, Arora A, Nain L (2013). Novel perspectives for evolving enzyme cocktails for lignocellulose hydrolysis in biorefineries. Sustain Chem Process.

[16] Jiménez DJ, Korenblum E, van Elsas JD (2014). Novel multi-species microbial consortia involved in lignocellulose and 5-hydroxymethylfurfural bioconversion. Appl Microbiol Biotechnol.

[17] Szabó KE, Itor P, Bertilsson S, Tranvik L, Eiler A (2007). Importance of rare and abundant populations for the structure and functional potential of freshwater bacterial communities. Aquat Microb Ecol.

[18] Elshahed M, Youssef NH, Spain AM, Sheik C, Najar FZ, Sukharnikov LO, Roe B, Davis JP, Schloss PD, Bailey VL, Krumholz LR (2008). Novelty and uniqueness patterns of rare members of the soil biosphere. Appl Environ Microbiol.

[19] Lynch MDJ, Neufeld JD (2015). Ecology and exploration of the rare biosphere. NatRev Microbiol.

[20] Jousset A, Bienhold C, Chatzinotas A, Gallien L, Gobet A, Kurm V, Küsel K, Rillig MC, Rivett DW, Salles JF, van der Heijden MG, Youssef NH, Zhang X, Wei Z (2017). Where less may be more: how the rare biosphere pulls ecosystems strings. ISME J.

[21] Pedrós-Alió C (2007). Dipping into the rare biosphere. Science.

[22] Reid A, Buckley M (2011). The rare biosphere.

[23] Sogin ML, Morrison HG, Huber JA, Mark Welch D, Huse SM, Neal PR, Arrieta JM, Herndl GJ (2006). Microbial diversity in the deep sea and the underexplored “rare biosphere”. Proc Natl Acad Sci.

[24] Campbell BJ, Yu L, Heidelberg JF, Kirchman DL (2011). Activity of abundant and rare bacteria in a coastal ocean. Proc Natl Acad Sci.

[25] Jiménez DJ, Dini-Andreote F, van Elsas JD (2014). Metataxonomic profiling and prediction of functional behaviour of wheat straw degrading microbial consortia. Biotechnol Biofuels.

[26] Caporaso JG, Kuczynski J, Stombaugh J, Bittinger K, Bushman FD, Costello EK, Fierer N, Peña AG, Goodrich JK, Gordon JI, Huttley GA, Kelley ST, Knights D, Koenig JE, Ley RE, Lozupone CA, McDonald D, Muegge BD, Pirrung M, Reeder J, Sevinsky JR, Turnbaugh PJ, Walters WA, Widmann J, Yatsunenko T, Zaneveld J, Knight R (2010). QIIME allows analysis of high-throughput community sequencing data. Nat Methods.

[27] Edgar RC (2010). Search and clustering orders of magnitude faster than BLAST. Bioinformatics.

[28] DeSantis TZ, Hugenholtz P, Larsen N, Rojas M, Brodie EL, Keller KT, Huber T, Dalevi DP, Hu P, Andersen GL (2006). Greengenes, a chimera-checked 16S rRNA gene database and workbench compatible with ARB. Appl Environ Microbiol.

[29] Balvočiūtė M, Huson DH (2017). SILVA, RDP, Greengenes, NCBI and OTT—how do these taxonomies compare?. BMC Genomics.

[30] Yan W, Ma H, Shi G, Li Y, Sun B, Xiao X, Zhang Y (2017). Independent shifts of abundant and rare bacterial populations across East Antarctica glacial foreland. Front Microbiol.

[31] Pedrós-Alió C (2006). Marine microbial diversity: can it be determined?. Trends Microbiol.

[32] Adapa PK, Tabil LG, Schoenau GJ, Canam T, Dumonceaux T (2011). Quantitative analysis of lignocellulosic components of non-treated and steam exploded barley, canola, oat and wheat straw using Fourier transform infrared spectroscopy. J Agr Sci Tech Issue.

[33] ter Braak, CJF, Šmilauer, P (2012) Canoco reference manual and user’s guide: software for ordination, version 50 Microcomputer Power, Ithaca

[34] Canilha L, Chandel AK, dos Santos Milessi TS, Fernandes Antunes FA, da Costa Freitas WL, Almeida Felipe M, Silvério da Silva S (2012). Bioconversion of sugarcane biomass into ethanol: an overview about composition, pretreatment methods, detoxification of hydrolysates, enzymatic saccharification, and ethanol fermentation. J Biomed Biotechnol.

[35] Leps J, Smilauer P (2003). Multivariate analysis of ecological data using CANOCO.

[36] Salles JF (2004). Multivariate analyses of Burkholderia species in soil: effect of crop and land use history. Appl Environ Microbiol.

[37] Delgado-Baquerizo M, Oliverio AM, Brewer TE, Benavent-González A, Eldridge DJ, Bardgett RD, Maestre FT, Singh BK, Fierer N (2018). A global atlas of the dominant bacteria found in soil. Science.

[38] Kishi LT, Lopes EM, Fernandes CC, Fernandes GC, Sacco LP, Carareto Alves LM, Lemos EGM (2017). Draft genome sequence of a *Chitinophaga* strain isolated from a lignocellulose biomass-degrading consortium. Genome Announc.

[39] Chandra R, Abhishek A, Sankhwar M (2011). Bacterial decolorization and detoxification of black liquor from rayon grade pulp manufacturing paper industry and detection of their metabolic products. Bioresour Technol.

[40] Jiménez DJ, de Lima Brossi MJ, Schückel J, Kračun SK, Willats WGT, van Elsas JD (2016). Characterization of three plant biomass-degrading microbial consortia by metagenomics- and metasecretomics-based approaches. Appl Microbiol Biotechnol.

[41] DeAngelis KM, D’Haeseleer P, Chivian D, Fortney JL, Khudyakov J, Simmons B, Woo H, Arkin AP, Davenport KW, Goodwin L, Chen A, Ivanova N, Kyrpides NC, Mavromatis K, Woyke T, Hazen TC (2011). Complete genome sequence of *Enterobacter lignolyticus SCF1*. Stand Genomic Sci.

[42] Sharmin F, Wakelin S, Huygens F, Hargreaves M (2013). Firmicutes dominate the bacterial taxa within sugar-cane processing plants. Sci Rep.

[43] Masai E, Katayama Y, Fukuda M (2007). Genetic and biochemical investigations on bacterial catabolic pathways for lignin-derived aromatic compounds. Biosci Biotechnol Biochem.

[44] Wang Y, Liu Q, Yan L, Gao Y, Wang Y, Wang W (2013). A novel lignin degradation bacterial consortium for efficient pulping. Bioresour Technol.

[45] Duan J, Liang D, Du WJ, Wang DQ (2014). Biodegradation of kraft lignin by a bacterial strain *Sphingobacterium sp HY-H*. Ad Mat Res.

[46] Jiménez DJ, Chaves-Moreno D, van Elsas JD (2015). Unveiling the metabolic potential of two soil-derived microbial consortia selected on wheat straw. Sci Rep.

[47] Ventorino V, Aliberti A, Faraco V, Robertiello A, Giacobbe S, Ercolini D, Amore A, Fagnano M, Pepe O (2015). Exploring the microbiota dynamics related to vegetable biomasses degradation and study of lignocellulose-degrading bacteria for industrial biotechnological application. Sci Rep.

[48] McBride MJ, Xie G, Martens EC, Lapidus A, Henrissat B, Rhodes RG, Goltsman E, Wang W, Xu J, Hunnicutt DW, Staroscik AM, Hoover TR, Cheng YQ, Stein JL (2009). Novel features of the polysaccharide-digesting gliding bacterium *Flavobacterium johnsoniae* as revealed by genome sequence analysis. Appl Environ Microbiol.

[49] DeAngelis KM, Gladden JM, Allgaier M, D’haeseleer P, Fortney JL, Reddy A, Hugenholtz P, Singer SW, Gheynst JSV, Silver WL, Simmons BA, Hazen TC (2010). Strategies for enhancing the effectiveness of metagenomic-based enzyme discovery in lignocellulolytic microbial communities. Bioenerg Res.

[50] Hottes AK, Meewan M, Yang D, Arana N, Romero P, McAdams HH, Stephens C (2004). Transcriptional profiling of *Caulobacter crescentus* during growth on complex and minimal media. J Bacteriol.

[51] Quinteros R, Goodwin S, Lenz RW, Park WH (1999). Extracellular degradation of medium chain length poly(beta-hydroxyalkanoates) by *Comamonas sp*. Int J Biol Macromol.

[52] Franzenburg S, Walter J, Künzel S, Wang J, Baines JF, Bosch TC, Fraune S (2013). Distinct antimicrobial peptide expression determines host species-specific bacterial associations. Proc Natl Acad Sci U S A.

[53] Kurth D, Romero CM, Fernandez PM, Ferrero MA, Martinez MA (2016). Draft genome sequence of *Achromobacter sp* strain AR476-2, isolated from a cellulolytic consortium. Genome Announc.

[54] Peng G, Zhu W, Wang H, Lü Y, Wang X, Zheng D, Cui Z (2010). Functional characteristics and diversity of a novel lignocelluloses degrading composite microbial system with high xylanase activity. J Microbiol Biotechnol.

[55] Yang H, Wu H, Wang X, Cui Z, Li Y (2011). Selection and characteristics of a switchgrass-colonizing microbial community to produce extracellular cellulases and xylanases. Bioresour Technol.

[56] Flint H, Scott K, Duncan S, Louis P, Forano E (2012). Microbial degradation of complex carbohydrates in the gut Gut. Microbes.

[57] Martinez-Garcia M, Brazel DM, Swan BK, Arnosti C, Chain PS, Reitenga KG, Xie G, Poulton NJ, Lluesma Gomez M, Masland DE, Thompson B, Bellows WK, Ziervogel K, Lo CC, Ahmed S, Gleasner CD, Detter CJ, Stepanauskas R (2012). Capturing single cell genomes of active polysaccharide degraders: an unexpected contribution of *Verrucomicrobia*. PLoS One.

[58] Romano N, Gioffré A, Sede SM, Campos E, Cataldi A, Talia P (2013). Characterization of cellulolytic activities of environmental bacterial consortia from an Argentinian native forest. Curr Microbiol.

[59] Liao HH, Zhang XZ, Rollin JA, Zhang Y-HP (2011). A minimal set of bacterial cellulases for consolidated bioprocessing of lignocellulose. Biotechnol J.

