# Supplementary material for: Dynamics of Abundant and Rare Bacteria During Degradation of Lignocellulose from Sugarcane Biomass
Source: Microb Ecol. 2019 Jul 8;79(2):312–25. doi: 10.1007/s00248-019-01403-w (PMC7033055; doi:10.1007/s00248-019-01403-w)
Supplement: Supplementary file 1 — (PPTX 786 kb) [file 248_2019_1403_MOESM1_ESM.pptx]

## Slide 1
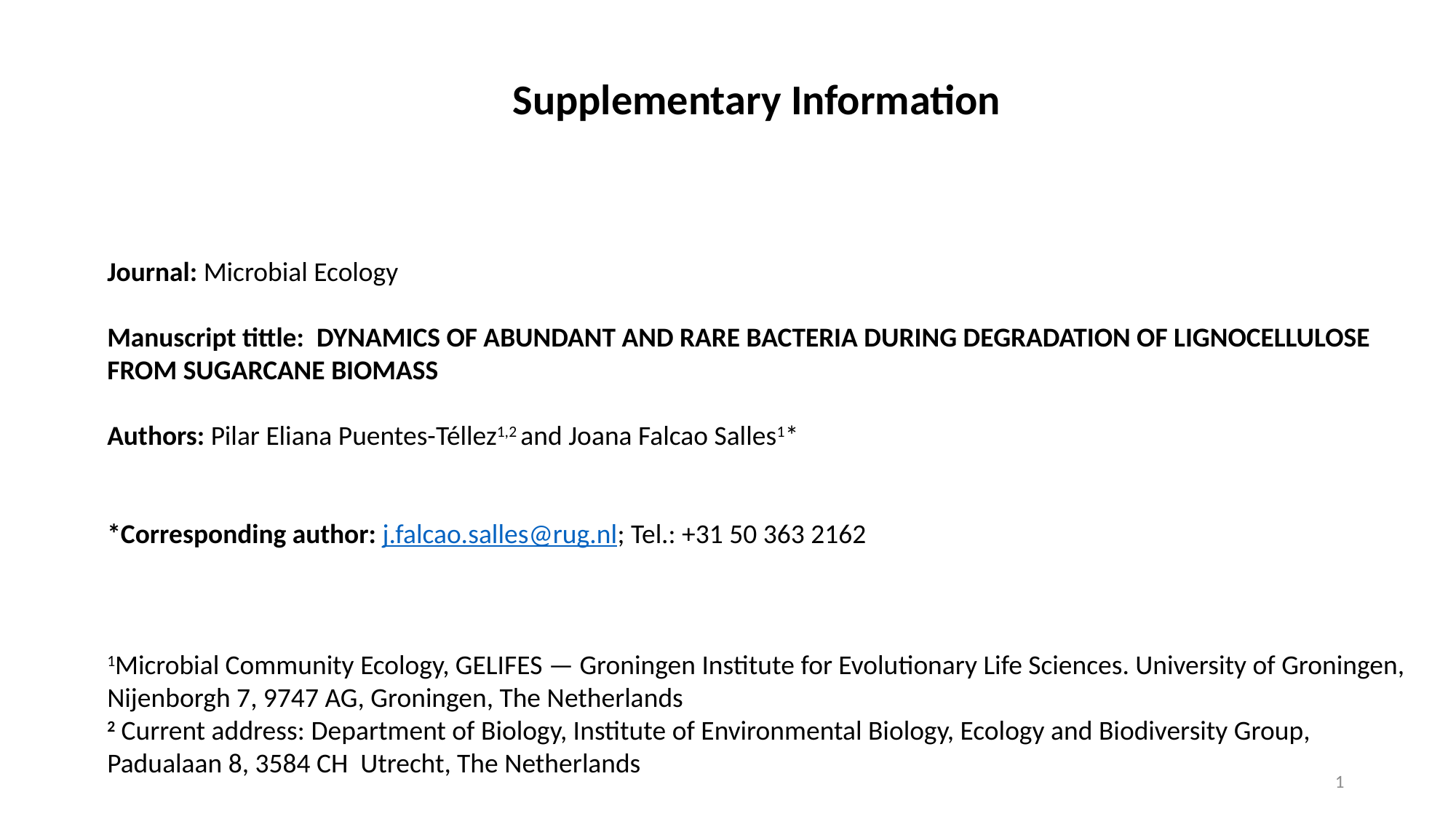

Supplementary Information
Journal: Microbial Ecology
Manuscript tittle:  DYNAMICS OF ABUNDANT AND RARE BACTERIA DURING DEGRADATION OF LIGNOCELLULOSE FROM SUGARCANE BIOMASS
Authors: Pilar Eliana Puentes-Téllez1,2 and Joana Falcao Salles1*
*Corresponding author: j.falcao.salles@rug.nl; Tel.: +31 50 363 2162
1Microbial Community Ecology, GELIFES — Groningen Institute for Evolutionary Life Sciences. University of Groningen, Nijenborgh 7, 9747 AG, Groningen, The Netherlands
2 Current address: Department of Biology, Institute of Environmental Biology, Ecology and Biodiversity Group, Padualaan 8, 3584 CH Utrecht, The Netherlands
1

## Slide 2
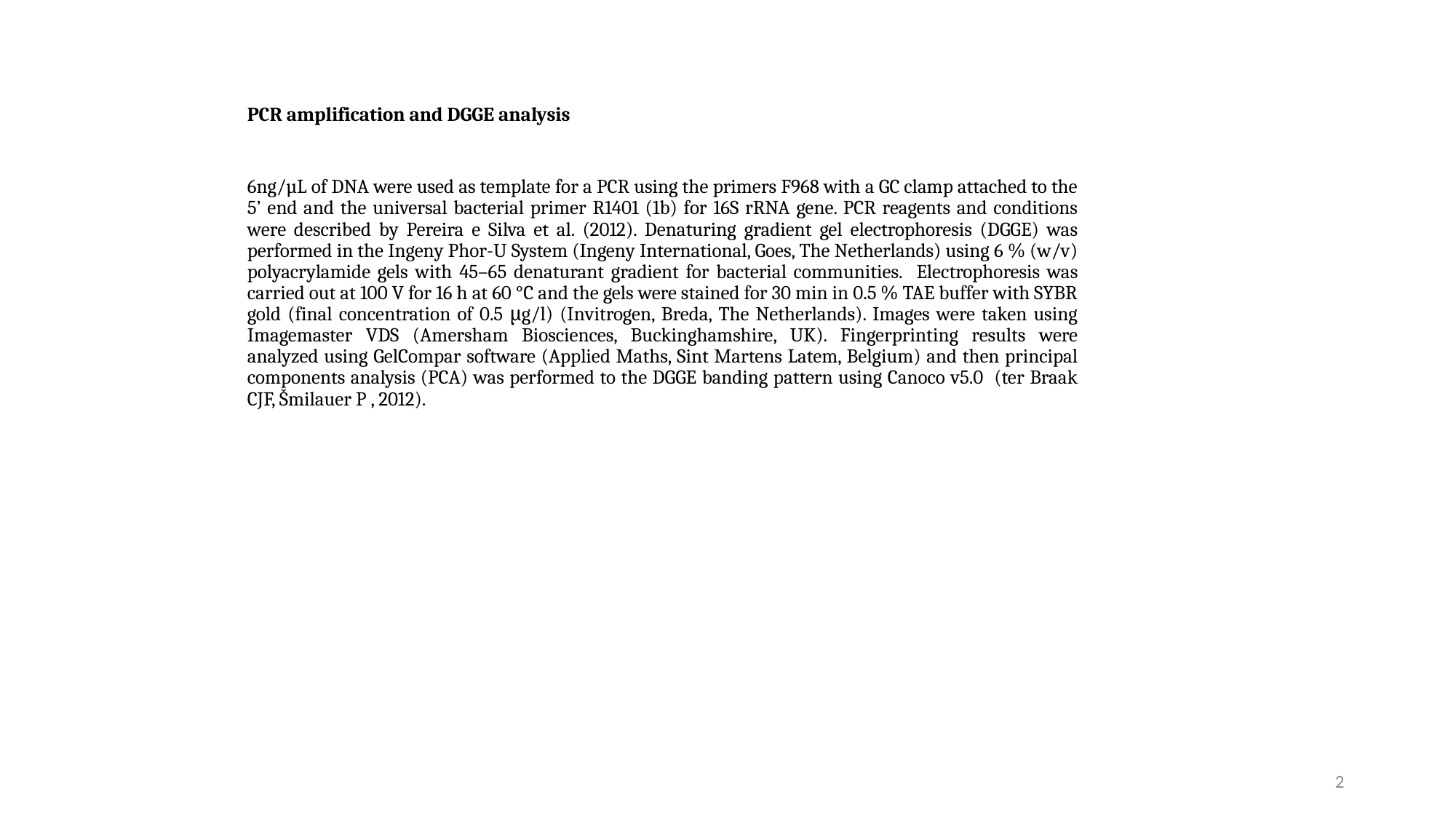

PCR amplification and DGGE analysis
6ng/µL of DNA were used as template for a PCR using the primers F968 with a GC clamp attached to the 5’ end and the universal bacterial primer R1401 (1b) for 16S rRNA gene. PCR reagents and conditions were described by Pereira e Silva et al. (2012). Denaturing gradient gel electrophoresis (DGGE) was performed in the Ingeny Phor-U System (Ingeny International, Goes, The Netherlands) using 6 % (w/v) polyacrylamide gels with 45–65 denaturant gradient for bacterial communities. Electrophoresis was carried out at 100 V for 16 h at 60 °C and the gels were stained for 30 min in 0.5 % TAE buffer with SYBR gold (final concentration of 0.5 μg/l) (Invitrogen, Breda, The Netherlands). Images were taken using Imagemaster VDS (Amersham Biosciences, Buckinghamshire, UK). Fingerprinting results were analyzed using GelCompar software (Applied Maths, Sint Martens Latem, Belgium) and then principal components analysis (PCA) was performed to the DGGE banding pattern using Canoco v5.0 (ter Braak CJF, Šmilauer P , 2012).
2

## Slide 3
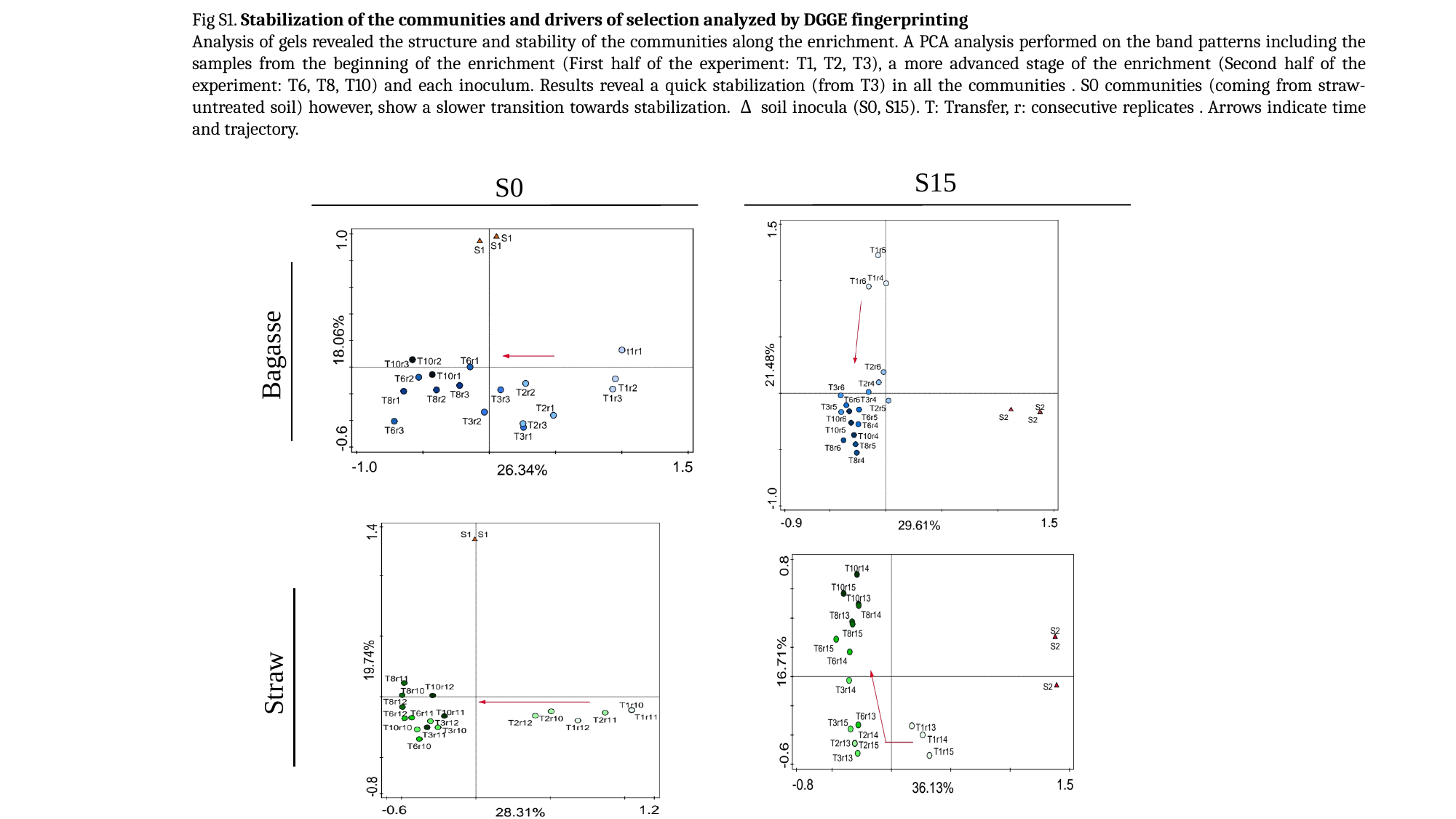

Fig S1. Stabilization of the communities and drivers of selection analyzed by DGGE fingerprinting
Analysis of gels revealed the structure and stability of the communities along the enrichment. A PCA analysis performed on the band patterns including the samples from the beginning of the enrichment (First half of the experiment: T1, T2, T3), a more advanced stage of the enrichment (Second half of the experiment: T6, T8, T10) and each inoculum. Results reveal a quick stabilization (from T3) in all the communities . S0 communities (coming from straw-untreated soil) however, show a slower transition towards stabilization.  Δ soil inocula (S0, S15). T: Transfer, r: consecutive replicates . Arrows indicate time and trajectory.
S15
S0
Bagasse
Straw

## Slide 4
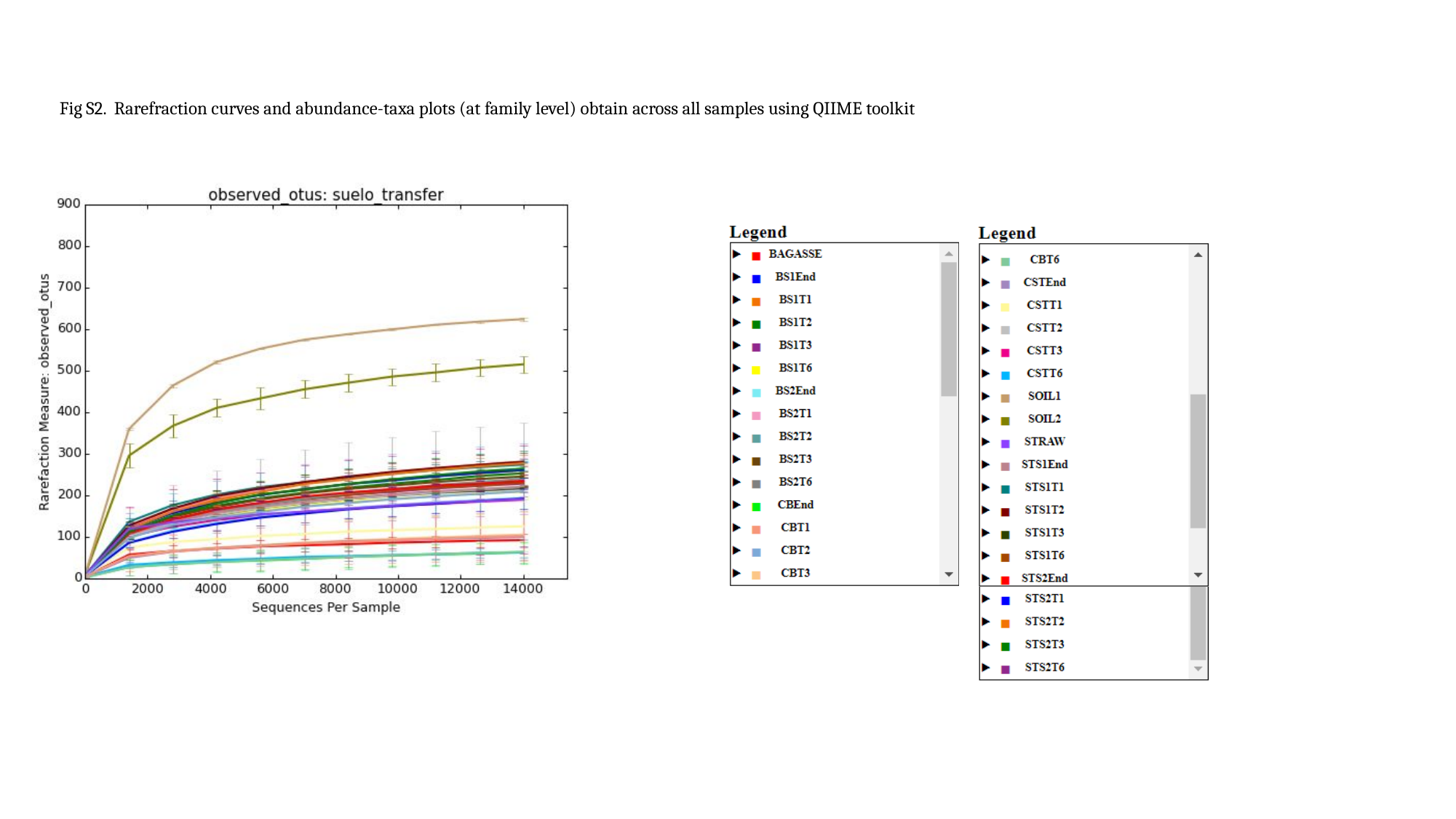

Fig S2. Rarefraction curves and abundance-taxa plots (at family level) obtain across all samples using QIIME toolkit

## Slide 5
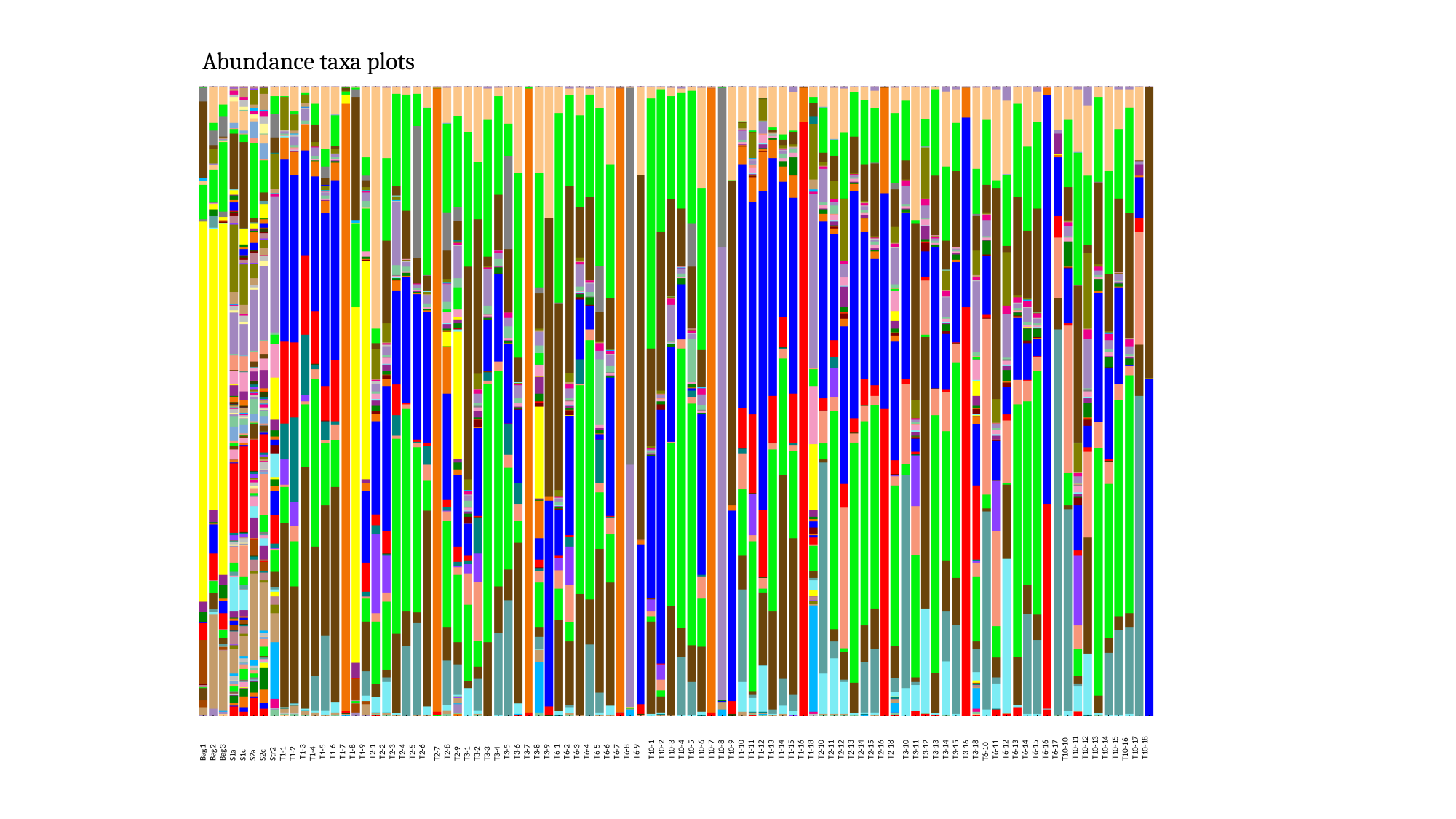

Abundance taxa plots
Bag1
Bag2
Bag3
S1a
S1c
S2a
S2c
Str2
T1-1
T1-2
 T1-3
T1-4
 T1-5
 T1-6
 T1-7
 T1-8
 T1-9
 T2-1
 T2-2
 T2-3
 T2-4
 T2-5
 T2-6
T2-7
 T2-8
T2-9
T3-1
T3-2
T3-3
T3-4
 T3-5
 T3-6
 T3-7
 T3-8
 T3-9
 T6-1
 T6-2
 T6-3
 T6-4
 T6-5
 T6-6
 T6-7
 T6-8
 T6-9
 T10-1
 T10-2
 T10-3
 T10-4
 T10-5
 T10-6
 T10-7
 T10-8
 T10-9
 T1-10
 T1-11
 T1-12
 T1-13
 T1-14
 T1-15
 T1-16
 T1-18
 T2-10
 T2-11
 T2-12
 T2-13
 T2-14
 T2-15
 T2-16
 T2-18
 T3-10
 T3-11
 T3-12
 T3-13
 T3-14
 T3-15
 T3-16
 T3-18
T6-10
 T6-11
 T6-12
 T6-13
 T6-14
 T6-15
 T6-16
 T6-17
T10-10
 T10-11
 T10-12
 T10-13
 T10-14
 T10-15
T10-16
 T10-17
 T10-18

## Slide 6
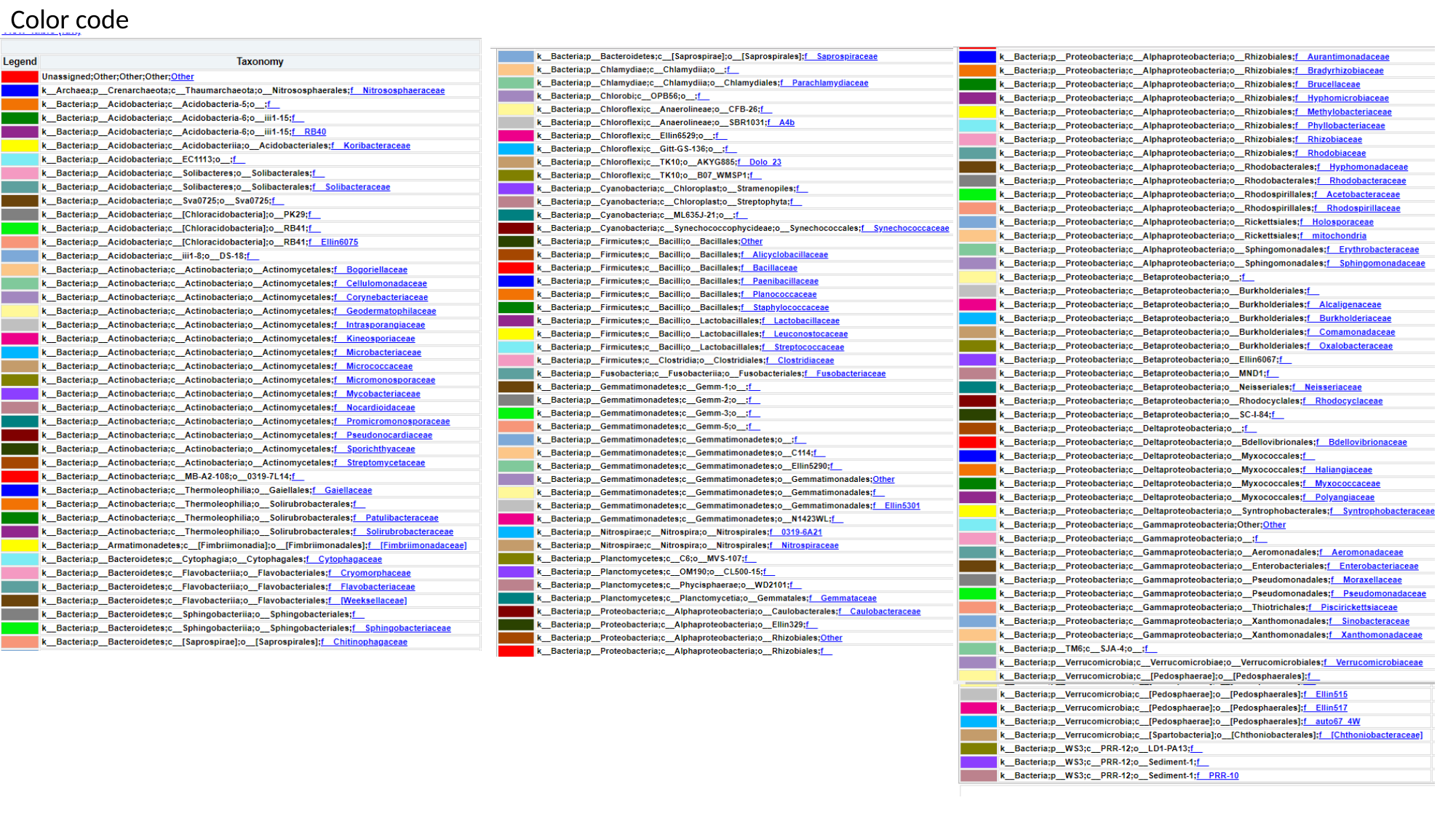

Color code

## Slide 7
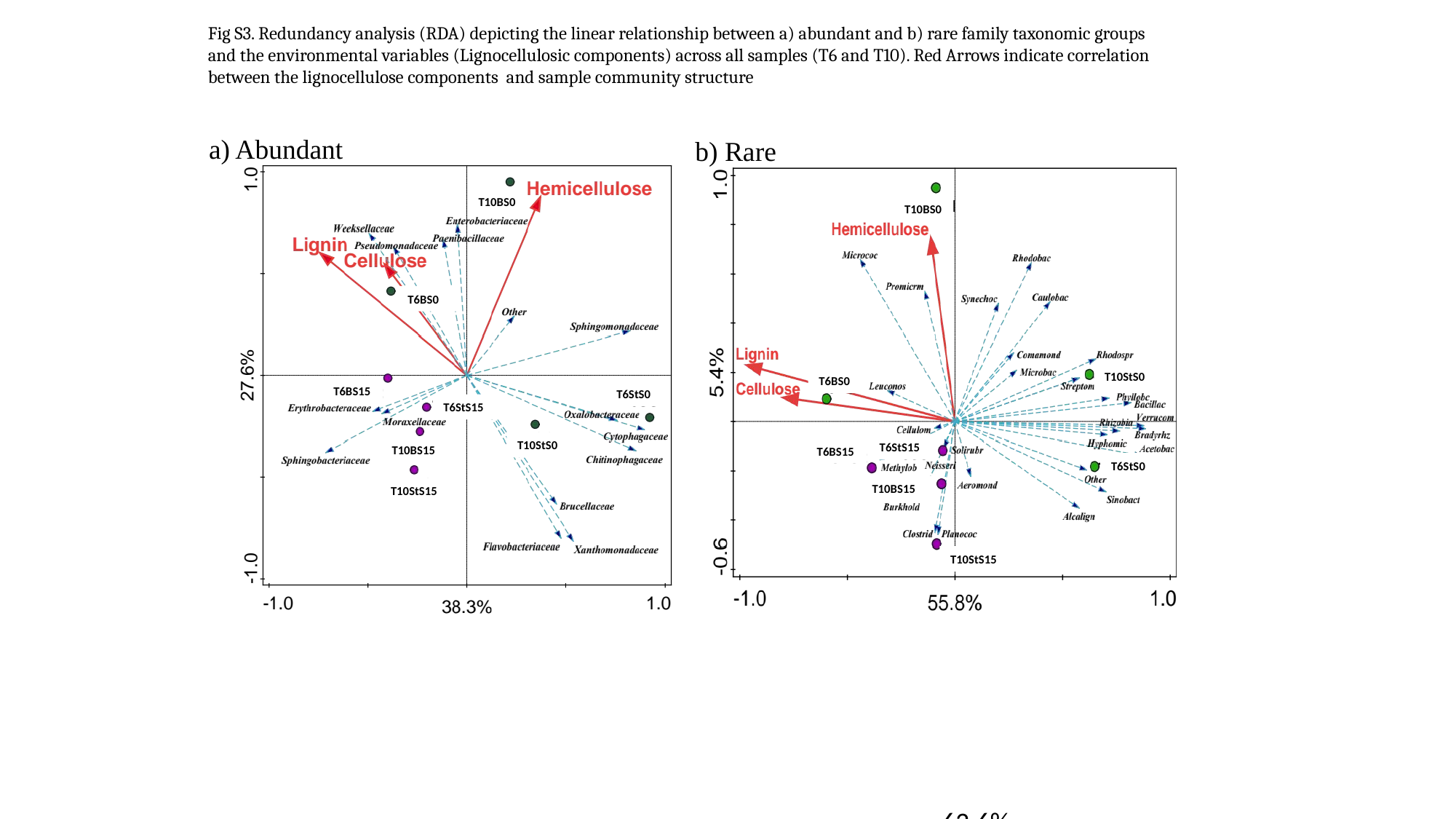

Fig S3. Redundancy analysis (RDA) depicting the linear relationship between a) abundant and b) rare family taxonomic groups and the environmental variables (Lignocellulosic components) across all samples (T6 and T10). Red Arrows indicate correlation between the lignocellulose components and sample community structure
a) Abundant
b) Rare
T10BS0
T10BS0
T6BS0
T10StS0
T6BS0
T6BS15
T6StS0
T6StS15
T10StS0
T6StS15
T10BS15
T6BS15
T6StS0
T10BS15
T10StS15
T10StS15
62.6%

## Slide 8
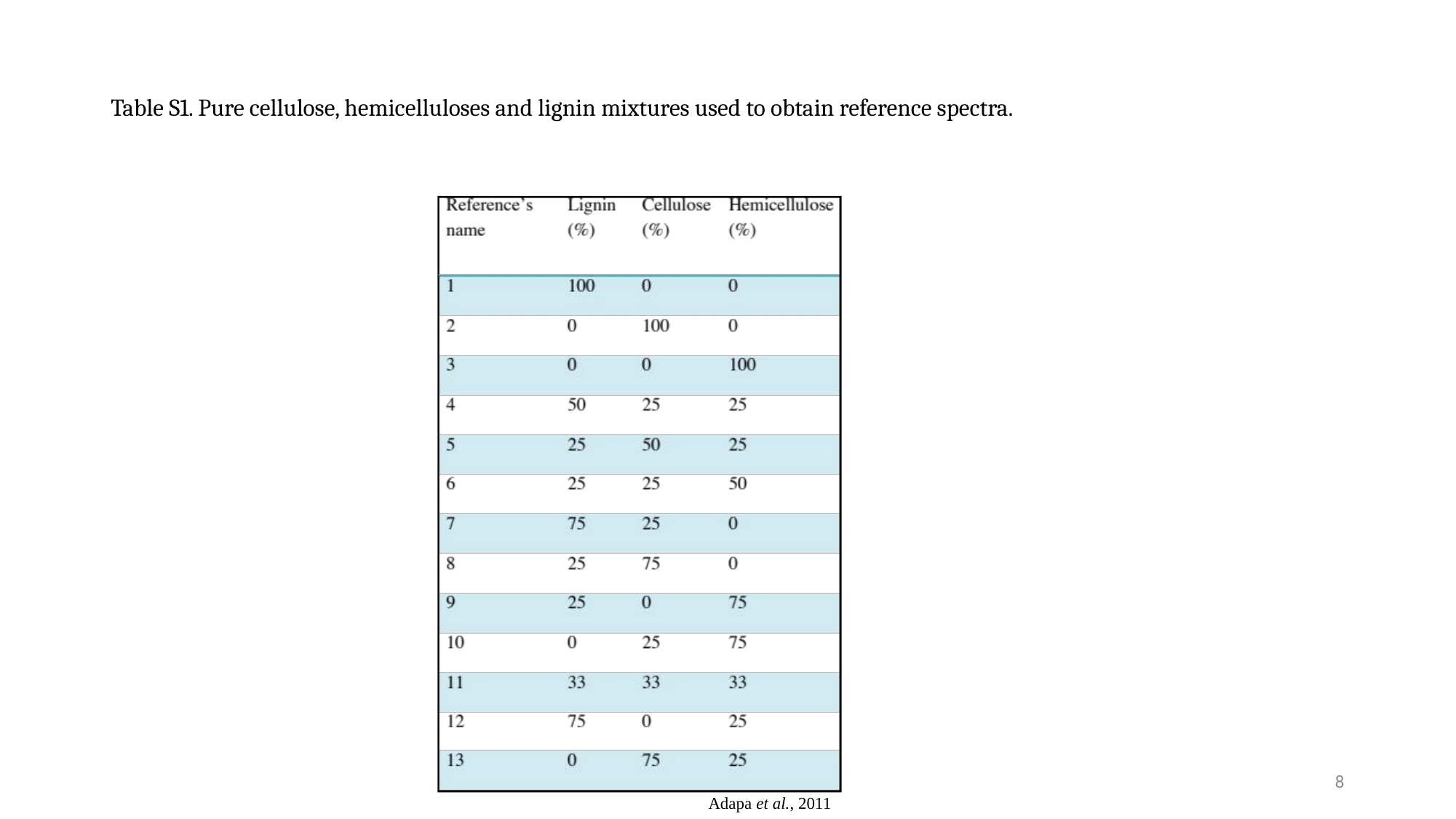

# Table S1. Pure cellulose, hemicelluloses and lignin mixtures used to obtain reference spectra.
8
Adapa et al., 2011

## Slide 9
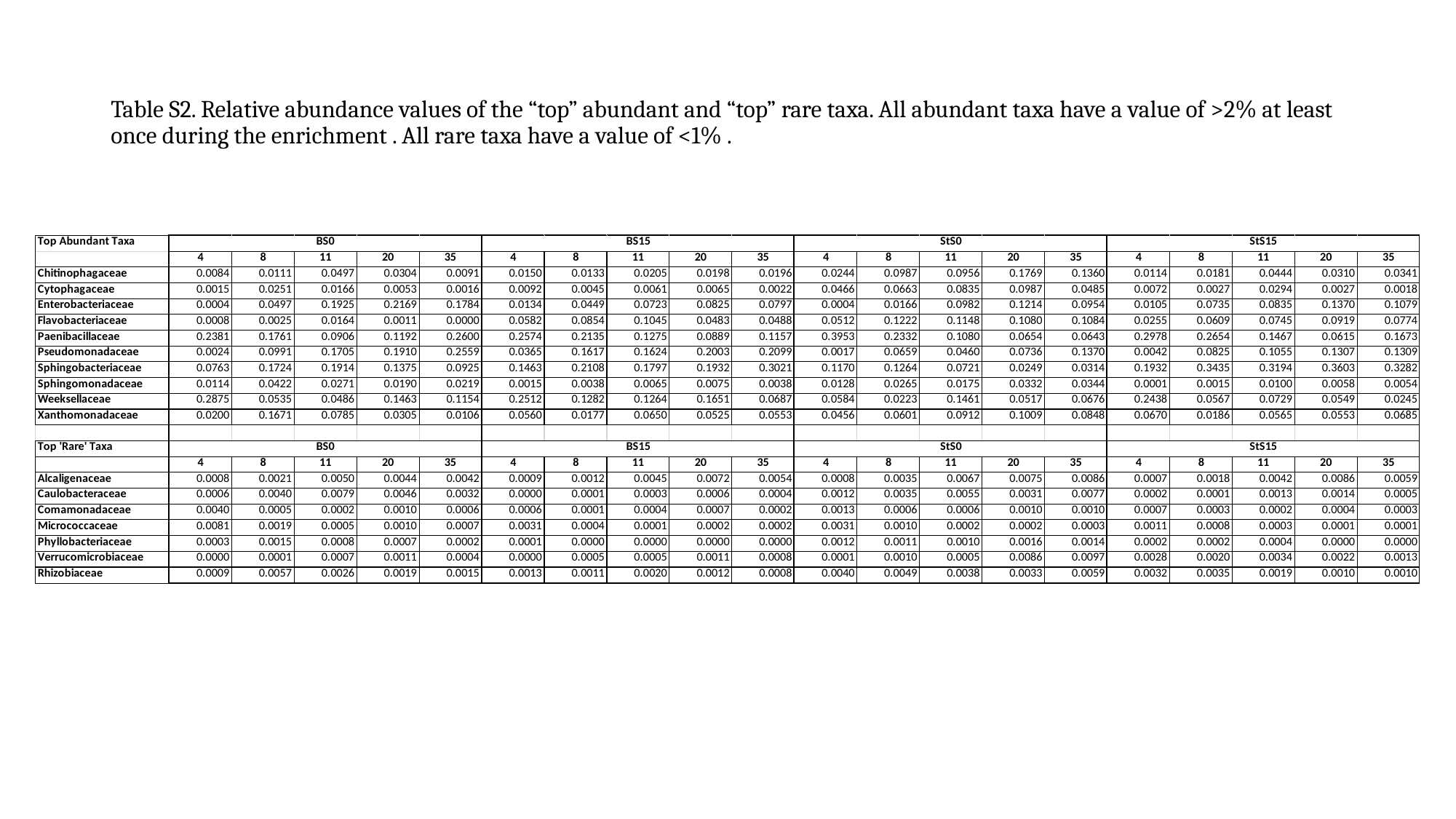

# Table S2. Relative abundance values of the “top” abundant and “top” rare taxa. All abundant taxa have a value of >2% at least once during the enrichment . All rare taxa have a value of <1% .

## Slide 10
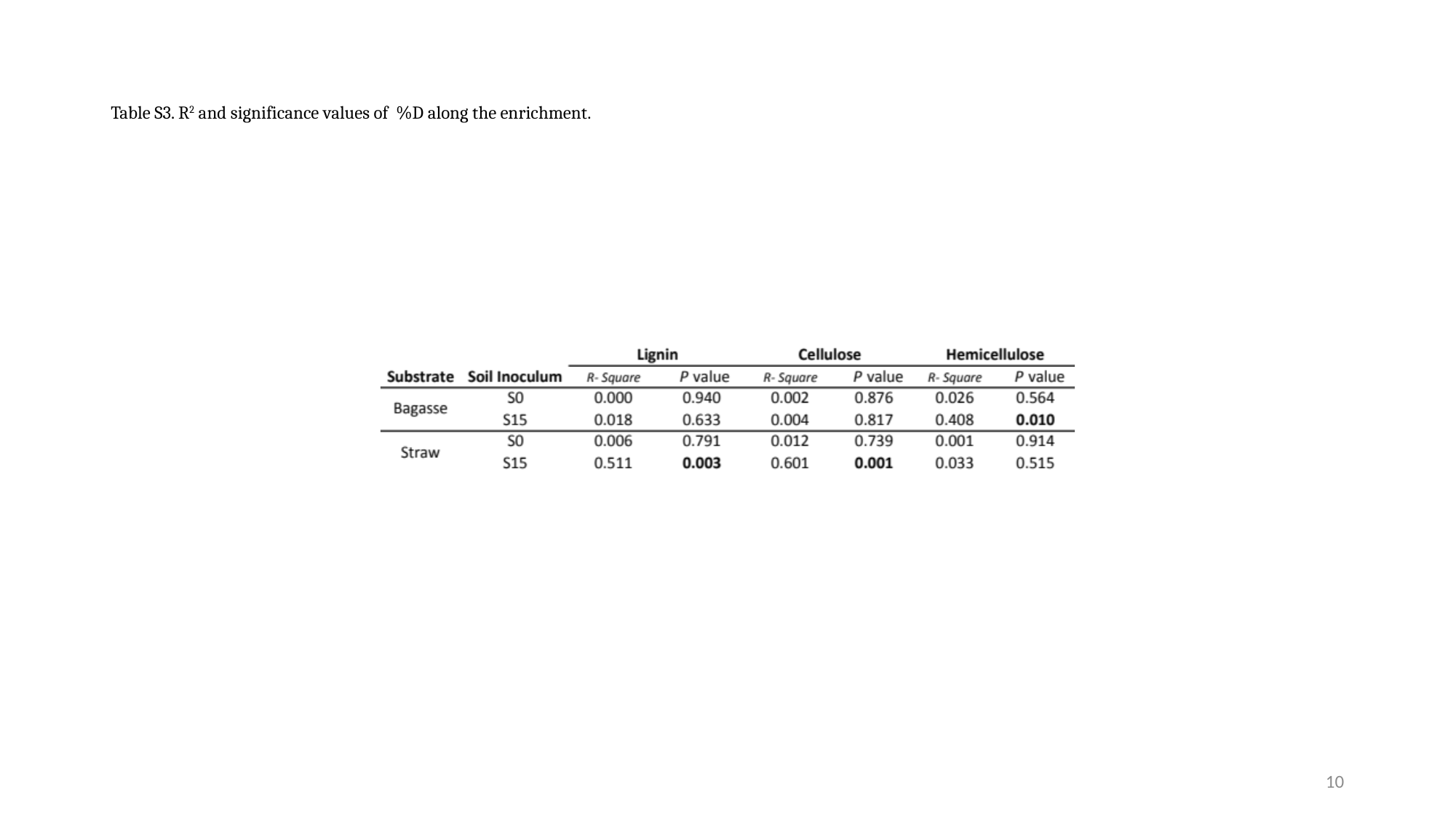

# Table S3. R2 and significance values of %D along the enrichment.
10

## Slide 11
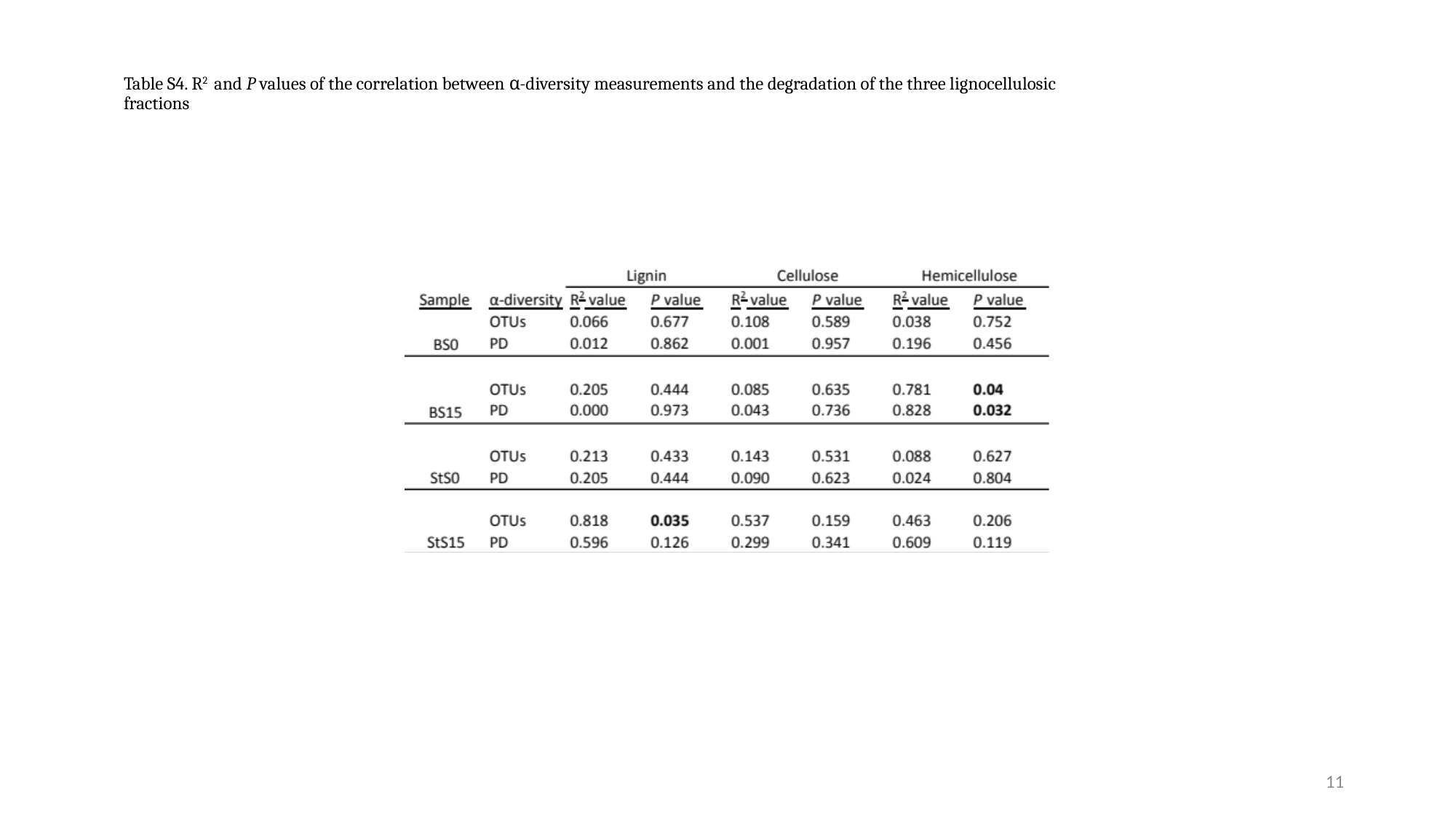

# Table S4. R2 and P values of the correlation between α-diversity measurements and the degradation of the three lignocellulosic fractions
11
